# Rapid detection of CITES-listed shark fin species by loop-mediated isothermal amplification assay with potential for field use

**DOI:** 10.1038/s41598-020-61150-8

**Published:** 2020-03-10

**Authors:** Grace Wing-Chiu But, Hoi-Yan Wu, Kwang-Tsao Shao, Pang-Chui Shaw

**Affiliations:** 1 School of Life Sciences, The Chinese University of Hong Kong, Shatin, Hong Kong SAR; 2 Institute of Chinese Medicine, The Chinese University of Hong Kong, Shatin, Hong Kong SAR; 3 Li Dak Sum Yip Yio Chin R & D Centre for Chinese Medicine, The Chinese University of Hong Kong, Shatin, Hong Kong SAR; 4 Systematics and Biodiversity Information Division, Biodiversity Research Center, Academia Sinica, Taipei, Taiwan

**Keywords:** PCR-based techniques, PCR-based techniques, Marine biology, Marine biology

## Abstract

Shark fin is a delicacy in many Asian countries. Overexploitation of sharks for shark fin trading has led to a drastic reduction in shark population. To monitor international trade of shark fin products and protect the endangered species from further population decline, we present rapid, user-friendly and sensitive diagnostic loop-mediated isothermal amplification (LAMP) and effective polymerase chain reaction (PCR) assays for all twelve CITES-listed shark species. Species-specific LAMP and PCR primers were designed based on cytochrome oxidase I (COI) and NADH2 regions. Our LAMP and PCR assays have been tested on 291 samples from 93 shark and related species. Target shark species could be differentiated from non-target species within three hours from DNA extraction to LAMP assay. The LAMP assay reported here is a simple and robust solution for on-site detection of CITES-listed shark species with shark fin products.

## Introduction

Shark fins are primarily consumed for their translucent collagen fibres served in Asian luxurious shark fin soup^1,2^. The global shark fin market was estimated to be worth USD $400–550 million annually and Hong Kong is one of the largest shark fin trade markets in the world, accounting for more than half of global trade volume^3–5^. Although the trade volume in Hong Kong has dropped by 30–50% since peaked at 2003 due to public campaign of shark population awareness, Hong Kong is still remaining a major market for fin trading with about 80 nations annually and the import volume of shark fins into Hong Kong was still over 4,500,000 kg in 2013, amounting USD $170 million^6–8^.

Sharks grow slowly and reach their sexual maturity late and they have a low fecundity and reproductive rate. Growing demand of shark fins and meat has led to overexploitation of shark, resulting in serious declines in some of the shark populations^9,10^. In order to avoid some shark species from overexploitation, the Convention on International Trade in Endangered Species of Wild Fauna and Flora (CITES) has listed twelve shark species in Appendix II, including, since 2000, great white shark (*Carcharodon carcharias*), basking shark (*Cetorhinus maximus*), and whale shark (*Rhincodon typus*); since 2013, oceanic whitetip shark (*Carcharhinus longimanus*), porbeagle shark (*Lamna nasus*), and three species of hammerhead sharks (*Sphyrna lewini*, *Sphyrna mokarran*, *Sphyrna zygaena*); since 2016, all three species of thresher sharks (*Alopias pelagicus*, *Alopias superciliosus*, *Alopias vulpinus*) and the silky shark (*Carcharhinus falciformis*). International trade of any part of the listed species must be accompanied by permits or licenses to certify that each specimen has been harvested sustainably and legally^11^. Shark species which were added to the list of CITES Appendix II since 2013 are especially commercially valuable and traded in large numbers^3,12^.

Species-level identification of shark fin products is critical for effective law enforcement and fisheries management. In order to enforce the CITES obligations and identify illegal shark fin trade, morphological identification or polymerase chain reaction (PCR)-based method is employed for identification of shark fin products traditionally^3,13^. Regular practices of molecular means for shark species identification include DNA barcoding^3,14,15^, mini-DNA barcoding for highly degraded DNA^16^, and species-specific amplification with PCR-based method^17–22^. These genetic approaches for identifying the species-of-origin of shark fins have been carried out since 1990s and they are effective for shark fin samples with different levels of processing. However, these current amplification approaches are time-consuming. The requirement of molecular expertise and sequencing facilities would also be a big hindrance for customs officials. There have also been no species-specific primers targeting the oceanic whitetip shark and the whale shark.

An easy-to-use, rapid, and effective diagnostic method is needed for species-level identification of all twelve CITES-listed shark species. Recently, loop-mediated isothermal amplification (LAMP), a rapid amplification technique using DNA polymerase with strand displacement activity at a single temperature, has been developed^23,24^. In LAMP reactions, two to three pairs of specific primers are designed to target four to six distinct regions of target DNA and generate loop-formed DNA, which increases sensitivity and specificity when compared to the conventional PCR methods^25^. Since it does not require a thermal cycler and can be detected by visual observation of fluorescence, it is favourable for field detection^26^. Recent studies have applied the LAMP technique on species-specific detection of animals^27,28^ and medicinal material^29,30^.

Most shark fins in Hong Kong are processed and classified in Chinese-name categories instead of species origin^3,12,31^. This presents difficulty in species-level identification for the law enforcement as well as for research and development of sustainable shark fisheries. Here, we present a rapid molecular method for on-site identification of shark fins based on species-specific amplification and instant detection for all twelve CITES-listed shark species. We have designed specific primers for both conventional species-specific PCR and LAMP methods based on cytochrome oxidase I (COI) and NADH dehydrogenase subunit 2 (NADH2) sequences. After sample preparation and DNA extraction, we are able to detect all twelve CITES-listed shark species individually within an hour at constant temperatures using these assays. Target shark species could be differentiated from non-target species within three hours from DNA extraction to LAMP assay.

## Materials and Methods

### Sources of samples and genetic identification of samples

In this project, 291 samples of 93 shark and related species from seven orders and 26 families were collected from the collection of The Chinese University of Hong Kong; and donations of Agriculture, Fisheries and Conservation Department of the HKSAR Government; Biodiversity Research Museum of Academia Sinica, Taiwan; Institute of Marine Biology of National Taiwan Ocean University; Kadoorie Farm & Botanic Garden Hong Kong; Ocean Park Hong Kong, and Ocean Park Conservation Foundation Hong Kong (Table 1). Among 291 samples, there were 94 dried processed fin samples, 174 frozen tissues, and 22 genomic DNA. For the 12 CITES-listed shark species, at least two samples were collected for each species. No live specimens were involved in the sample collection and the samples were either dry products from the market or donated by the various institutions. All samples, including dried processed fin samples, were stored at -80 °C until DNA extraction and downstream experiments.

**Table 1:** Shark and related samples for the validation of LAMP and PCR assay.

Order	Family	Species	Common name	Number of sample (unprocessed/ processed)
Carcharhiniformes	Carcharhinidae	*Carcharhinus acronotus*	Blacknose shark	0/1
Carcharhiniformes	Carcharhinidae	*Carcharhinus albimarginatus*	Silvertip shark	7/1
Carcharhiniformes	Carcharhinidae	*Carcharhinus altimus*	Bignose shark	3/2
Carcharhiniformes	Carcharhinidae	*Carcharhinus amblyrhynchoides*	Graceful shark	0/1
Carcharhiniformes	Carcharhinidae	*Carcharhinus amblyrhynchos*	Grey reef shark	0/1
Carcharhiniformes	Carcharhinidae	*Carcharhinus amboinensis*	Pigeye shark	0/1
Carcharhiniformes	Carcharhinidae	*Carcharhinus brachyurus*	Copper shark	4/3
Carcharhiniformes	Carcharhinidae	*Carcharhinus brevipinna*	Spinner shark	3/3
Carcharhiniformes	Carcharhinidae	Carcharhinus falciformis^*^	Silky shark	2/7
Carcharhiniformes	Carcharhinidae	*Carcharhinus galapagensis*	Galapagos shark	0/1
Carcharhiniformes	Carcharhinidae	*Carcharhinus isodon*	Finetooth shark	0/1
Carcharhiniformes	Carcharhinidae	*Carcharhinus leucas*	Bull shark	1/4
Carcharhiniformes	Carcharhinidae	*Carcharhinus limbatus*	Blacktip shark	5/2
Carcharhiniformes	Carcharhinidae	Carcharhinus longimanus^*^	Oceanic whitetip shark	2/5
Carcharhiniformes	Carcharhinidae	*Carcharhinus macloti*	Hardnose shark	0/1
Carcharhiniformes	Carcharhinidae	*Carcharhinus melanopterus*	Blacktip reef shark	1/2
Carcharhiniformes	Carcharhinidae	*Carcharhinus obscurus*	Dusky shark	1/2
Carcharhiniformes	Carcharhinidae	*Carcharhinus perezi*	Caribbean reef shark	0/1
Carcharhiniformes	Carcharhinidae	*Carcharhinus plumbeus*	Sandbar shark	4/2
Carcharhiniformes	Carcharhinidae	*Carcharhinus porosus*	Smalltail shark	0/1
Carcharhiniformes	Carcharhinidae	*Carcharhinus sorrah*	Spottail shark	1/6
Carcharhiniformes	Carcharhinidae	*Galeocerdo cuvier*	Tiger shark	2/4
Carcharhiniformes	Carcharhinidae	*Lamiopsis temminckii*	Broadfin shark	0/1
Carcharhiniformes	Carcharhinidae	*Loxodon macrorhinus*	Sliteye shark	0/1
Carcharhiniformes	Carcharhinidae	*Negaprion acutiden*	Sharptooth lemon shark	0/2
Carcharhiniformes	Carcharhinidae	*Negaprion brevirostris*	Lemon shark	0/1
Carcharhiniformes	Carcharhinidae	*Prionace glauca*	Blue shark	2/4
Carcharhiniformes	Carcharhinidae	*Rhizoprionodon acutus*	Milk shark	0/1
Carcharhiniformes	Carcharhinidae	*Rhizoprionodon longurio*	Pacific sharpnose shark	0/1
Carcharhiniformes	Carcharhinidae	*Rhizoprionodon taylori*	Australian sharpnose shark	0/1
Carcharhiniformes	Carcharhinidae	*Scoliodon laticaudus*	Spadenose shark	0/1
Carcharhiniformes	Hemigaleidae	*Hemigaleus australiensis*	Australian weasel shark	0/1
Carcharhiniformes	Hemigaleidae	*Hemipristis elongata*	Snaggletooth shark	0/1
Carcharhiniformes	Hemigaleidae	*Hemitriakis japanica*	Japanese topeshark	7/0
Carcharhiniformes	Pentanchidae	*Apristurus macrorhynchus*	Flathead catshark	3/0
Carcharhiniformes	Proscylliidae	*Proscyllium habereri*	Graceful catshark	7/0
Carcharhiniformes	Scyliorhinidae	*Apristurus nakayai*	Milk-eye catshark	1/0
Carcharhiniformes	Scyliorhinidae	*Apristurus platyrhynchus*	Borneo catshark	1/0
Carcharhiniformes	Scyliorhinidae	*Atelomycterus marmoratus*	Coral catshark	6/0
Carcharhiniformes	Scyliorhinidae	*Cephaloscyllium umbratile*	Blotchy swell shark	4/0
Carcharhiniformes	Scyliorhinidae	*Galeus sauteri*	Blacktip sawtail catshark	7/0
Carcharhiniformes	Scyliorhinidae	*Parmaturus xaniurus*	Filetail catshark	1/0
Carcharhiniformes	Sphyrnidae	*Eusphyra blochii*	Winghead shark	0/1
Carcharhiniformes	Sphyrnidae	Sphyrna lewini^*^	Scalloped hammerhead shark	1/6
Carcharhiniformes	Sphyrnidae	Sphyrna mokarran^*^	Great hammerhead shark	0/9
Carcharhiniformes	Sphyrnidae	*Sphyrna tiburo*	Bonnethead shark	0/1
Carcharhiniformes	Sphyrnidae	*Sphyrna tudes*	Smalleye hammerhead shark	0/1
Carcharhiniformes	Sphyrnidae	Sphyrna zygaena^*^	Smooth hammerhead shark	0/6
Carcharhiniformes	Triakidae	*Galeorhinus galeus*	Tope shark	0/1
Carcharhiniformes	Triakidae	*Mustelus californicus*	Gray smoothhound shark	0/1
Carcharhiniformes	Triakidae	*Mustelus canis*	Dusky smoothhound shark	0/1
Carcharhiniformes	Triakidae	*Mustelus griseus*	Spotless smoothhound shark	3/0
Carcharhiniformes	Triakidae	*Mustelus henlei*	Brown smoothhound shark	0/1
Carcharhiniformes	Triakidae	*Mustelus lunulatus*	Sicklefin smoothhound shark	0/1
Carcharhiniformes	Triakidae	*Mustelus manazo*	Starspotted smoothhound shark	9/0
Carcharhiniformes	Triakidae	*Mustelus mosis*	Arabian smoothhound shark	0/1
Carcharhiniformes	Triakidae	*Mustelus mustelus*	Common smoothhound shark	0/1
Carcharhiniformes	Triakidae	*Mustelus punctulatus*	Blackspotted smoothhound shark	0/1
Chimaeriformes	Callorhinchidae	*Callorhinchus callorynchus*	American elephantfish	0/1
Chimaeriformes	Chimaeridae	*Hydrolagus novaezealandiae*	Dark ghost shark	0/1
Hexanchiformes	Hexanchidae	*Heptranchias perlo*	Sharpnose sevengill shark	2/0
Hexanchiformes	Hexanchidae	*Hexanchus griseus*	Bluntnose sixgill shark	0/1
Lamniformes	Alopiidae	Alopias pelagicus^*^	Pelagic thresher shark	4/6
Lamniformes	Alopiidae	Alopias superciliosus^*^	Bigeye thresher shark	3/7
Lamniformes	Alopiidae	Alopias vulpinus^*^	Common thresher shark	0/2
Lamniformes	Cetorhinidae	Cetorhinus maximus^*^	Basking shark	0/5
Lamniformes	Lamnidae	Carcharodon carcharias^*^	Great white shark	2/1
Lamniformes	Lamnidae	*Isurus oxyrinchus*	Shortfin mako	10/6
Lamniformes	Lamnidae	*Isurus paucus*	Longfin mako	1/2
Lamniformes	Lamnidae	*Lamna ditropis*	Salmon shark	5/0
Lamniformes	Lamnidae	Lamna nasus^*^	Porbeagle	1/2
Lamniformes	Megachasmidae	*Megachasma pelagios*	Megamouth shark	4/0
Lamniformes	Mitsukurinidae	*Mitsukurina owstoni*	Goblin shark	1/0
Lamniformes	Odontaspididae	*Carcharias taurus*	Sand tiger shark	0/1
Orectolobiformes	Ginglymostomatidae	*Ginglymostoma cirratum*	Nurse shark	0/1
Orectolobiformes	Orectolobidae	*Orectolobus maculatus*	Spotted wobbegong	0/1
Orectolobiformes	Rhincodontidae	Rhincodon typus^*^	Whale shark	1/2
Squaliformes	Centrophoridae	*Centrophorus acus*	Gulper shark	2/0
Squaliformes	Centrophoridae	*Deania profundorum*	Arrowhead dogfish	0/1
Squaliformes	Centrophoridae	*Deania quadrispinosa*	Longsnout dogfish	1/0
Squaliformes	Dalatiidae	*Dalatias licha*	Kitefin shark	0/1
Squaliformes	Echinorhinidae	*Echinorhinus cookei*	Prickly shark	2/0
Squaliformes	Echinorhinidae	*Etmopterus brachyurus*	Shorttail lanternshark	5/0
Squaliformes	Echinorhinidae	*Etmopterus decacuspidatus*	Combtoothed lanternshark	1/0
Squaliformes	Echinorhinidae	*Etmopterus fusus*	Pygmy lanternshark	2/0
Squaliformes	Echinorhinidae	*Etmopterus molleri*	Mollers lantern shark	2/0
Squaliformes	Echinorhinidae	*Etmopterus pusillus*	Smooth lanternshark	4/0
Squaliformes	Etmopteridae	*Centroscyllium fabricii*	Black dogfish	4/0
Squaliformes	Etmopteridae	*Trigonognathus kabeyai*	Viper dogfish	1/0
Squaliformes	Somniosidae	*Somniosus pacificus*	Pacific sleeper shark	2/0
Squaliformes	Squalidae	*Squalus brevirostris*	Japanese shortnose spurdog	4/0
Squaliformes	Squalidae	*Squalus japonicus*	Japanese spurdog	4/0
Squatiniformes	Squatinidae	*Squatina legnota*	Indonesian angel shark	1/0

*CITES-listed shark species.

In order to ensure the species identity of the samples and avoid misidentification of samples due to other parties, DNA of all samples were extracted and amplified by PCR amplification. PCR products were sequenced, and the sequencing results were compared to the NCBI database using BLAST analysis. DNA was extracted from the samples using Biomed Genomic DNA/Tissue Extraction Kit (Biomed, Beijing, China) according to the manufacturer’s instructions (~60 minutes). PCR was performed to amplify COI gene using universal fish primers, FISHCOILBC_ts (5′-CACGACGTTGTAAAACGACTCAACYAATCAYAAAGATATYGGCAC-3′), FISHCOIHBC_ts (5′-GGATAACAATTTCACACAGGACTTCYGGGTGRCCRAARAATC-3′)^32^, FishF1 (5′- TCAACCAACCACAAAGACATTGGCAC-3′) and FishR1 (5′-TAGACTTCTGGGTGGCCAAAGAATCA-3′)^33^ for all the samples with an expected product size of ~700 bp. Sanger sequencing of PCR products purified with gel extraction kit (Biomed, Beijing, China) was performed by Tech Dragon Ltd., Hong Kong. Samples were identified using BLAST search against nucleotide sequences available on GenBank. The top-match results with a sequence similarity of at least 99% were used for species identification^34^. Sequences of COI, NADH2 and 12 S rRNA regions of sharks (12265, 1761, and 327 sequences respectively) were downloaded from the NCBI database for alignment. Species-specific primers were designed based on nucleotides unique for each of the 12 CITES-listed species.

### Design of species-specific LAMP primers and nucleic acid amplification assays using LAMP techniques

Thirteen sets of LAMP primers were designed using software PrimerExplorer V5 (Eiken Chemical Co. Ltd., Tokyo, Japan) and Primer-BLAST (https://www.ncbi.nlm.nih.gov/tools/primer-blast/). Each set contains at least four LAMP primers (external primers F3 and B3; forward and backward internal primers FIP and BIP), complementary to the species-specific sequences of the 12 target shark species or to the sequences conserved among all shark species as an internal control. Species-specific LAMP primers were designed based on the COI region for pelagic thresher shark, common thresher shark, great white shark, basking shark, whale shark, scalloped hammerhead shark, great hammerhead shark and smooth hammerhead shark. For bigeye thresher shark, silky shark, oceanic whitetip shark and porbeagle shark, the species-specific LAMP primers were designed based on the NADH2 region. Primers set for the internal control were based on the 12 S rRNA gene. For great white shark, porbeagle shark, great hammerhead shark and the internal control, loop primers (LF and/or LB) were added to the LAMP reactions. The sequences of all LAMP primers and the corresponding reaction conditions are shown in Table 2. LAMP reactions of target shark species were tested against all collected non-target species using the Isothermal Mastermix Amplification Kit (OptiGene, Horsham, UK) using Genie II (OptiGene, Horsham, UK) in triplicates. Each LAMP reaction mixture contained 1.0 μL of 10.0 ng/μL template DNA, 7.5 μL of Isothermal Mastermix, 0.5 μL of F3 (5 μmol/L), B3 (5 μmol/L), FIP (50 μmol/L), BIP (50 μmol/L), and, for great white shark, porbeagle shark, great hammerhead shark and the internal control, 0.5 μL of LF (25 μmol/L) and/or LB (25 μmol/L) with a final volume of 12.5 μL were applied as listed in Table 2. LAMP was performed at the corresponding temperature and reaction time (Table 2) using Genie II. For each run, positive and negative controls were included. DNA binding dye SYBR Green (2 μL of 1000X stock pre-diluted in distilled water) was added at the end of the reaction for naked-eye detection. With the amount of reagents and consumables used under this workflow, each LAMP reaction for one sample costs around USD $0.6. Time of experiment from DNA extraction to result analysis was approximately within 2–3 hours for 14 samples, one positive control and one negative control per run using Genie II.

**Table 2:** LAMP and PCR primers for CITES-listed shark detection.

Target	Region	Reaction	Primer Type	Primer Name	Sequence (5′ to 3′)	Reaction Temperature and Time	Amplicon Size (bp)
*Alopias pelagicus* (Pelagic thresher shark)	COI	LAMP	F3	AP02A	TTATACCCGTGATAATTGGC	70 °C for 60 min	296
			B3	AP03B	TGATAGTTGTAATAAAGTTG		
			FIP	AP2A3B FIPV2	GAGGGGGGAAGGAGTCAAAAGGATTTGGAAACTGACTAGTGC		
			BIP	AP2A3B BIPV9	GGAGCCGGTACTGGTTGAACCCTGCTAAATGGAGAGAGAA		
		PCR	LP	AP02A	TTATACCCGTGATAATTGGC	94 °C for 5 min; 35 cycles of 94 °C for 30 s, 58 °C for 30 s, and 72 °C for 1 min; 72 °C for 5 min	
			RP	AP03B	TGATAGTTGTAATAAAGTTG		
*Alopias superciliosus* (Bigeye thresher shark)	COI	LAMP	F3	AS10A	GTTGACTTGGCCATTTTCTCG	65 °C for 60 min	211
			B3	AS11B	CGATCAGTTAATAATATTGTG		
			FIP	AS10A11B FIPV2	GTTGTAATAAAGTTAATTGAAGCTCTTCATTTAGCAGGTATC		
			BIP	AS10A11B BIPV2	TCAAACACCATTATTTGTATGATCACTGGGAGGGATAAGAGGA		
		PCR	LP	AS10A	GTTGACTTGGCCATTTTCTCG	94 °C for 5 min; 35 cycles of 94 °C for 30 s, 62 °C for 30 s, and 72 °C for 1 min; 72 °C for 5 min	
			RP	AS11B	CGATCAGTTAATAATATTGTG		
*Alopias vulpinus* (Common thresher shark)	NADH2	LAMP	F3	AV02A	AATTGGCCTAGCCCCACTT	65 °C for 60 min	179
			B3	AV03B	CCTACTATAGTTGATAGTACTCCT		
			FIP	AV2A3B FIPV2	TCTAAGCCTTGGAGAACTTCCACTTCTGATTACCCGAA		
			BIP	AV2A3B BIPV2	TACTACCGGCCTCATTCTTTGTTTAGTGAAGGGTAAAGTTGT		
		PCR	LP	AV02A	AATTGGCCTAGCCCCACTT	94 °C for 5 min; 35 cycles of 94 °C for 30 s, 60 °C for 30 s, and 72 °C for 1 min; 72 °C for 5 min	
			RP	AV03B	CCTACTATAGTTGATAGTACTCCT		
*Carcharodon carcharias* (Great white shark)	COI	LAMP	F3	CC05A	TCTTCATGGTAATGCCCATC	70 °C for 50 min	236
			B3	CC04B	CCAGGTCAACGGATGCTCCT		
			FIP	CC5A4B FIPV1	TATGTTATTTATTCGGGGGAAGGCGGGAATTGACTAATCCCGTTAA		
			BIP	CC5A4B BIPV4	CTAGCTTCAGCCGGAGTTGAGTGCTAAATTACCGGCCAG		
			LF	CC5A4B F1B4 LF V1	TGTCCGGGGCACCAATTA		
			LB	CC5A4B F1B4 LBV1	GGAGCCGGCACTGGTTGAA		
		PCR	LP	CC05A	TCTTCATGGTAATGCCCATC	94 °C for 5 min; 35 cycles of 94 °C for 30 s, 66 °C for 30 s, and 72 °C for 1 min; 72 °C for 5 min	
			RP	CC04B	CCAGGTCAACGGATGCTCCT		
*Carcharhinus falciformis* (Silky shark)	NADH2	LAMP	F3	CF04A	TCACCACAGGACTTATCTTG	70 °C for 60 min	270
			B3	CF07B	GTAAGTGTTATGATAATGTAC		
			FIP	CF4A7B FIPV5	TCCTCATCCTCCAATGATTGTTGAGGCCCCATTTGCTATTTTGC		
			BIP	CF4A7B BIPV5	TTCTAGCCTACTCATCAATCGCAGGATGAGGTTAAGTAGGGTC		
	COI	PCR	LP	CF01A	TCTTCTAATTCGAGCTGAG	94 °C for 5 min; 35 cycles of 94 °C for 30 s, 64 °C for 30 s, and 72 °C for 1 min; 72 °C for 5 min	383
			RP	CF01B	ATTGAAGCTAGAATAGATGAC		
*Carcharhinus longimanus* (Oceanic whitetip shark)	NADH2	LAMP	F3	CL02A	CTCCTCTCCCTAGGAGGATTG	70 °C for 60 min	302
			B3	CL05B	TGGGATGAGTGTAAAGATTGC		
			FIP	CL2A5B FIPV1	GAGGGCTATAATAGTAGCTGGAGTACCTCCACTCTCCGGATTC		
			BIP	CL2A5B BIPV5	TGCTACAACACTAACTATAAGCCCCAGGAGGATAGATAAGGTTGC		
		PCR	LP	CL02A	CTCCTCTCCCTAGGAGGATTG	94 °C for 5 min; 35 cycles of 94 °C for 30 s, 68 °C for 30 s, and 72 °C for 1 min; 72 °C for 5 min	
			RP	CL05B	TGGGATGAGTGTAAAGATTGC		
*Cetorhinus maximus* (Basking shark)	COI	LAMP	F3	CM01A	ACCCGGATCACTTCTTGGT	68 °C for 60 min	530
			B3	CM02B	AAGAATGTTGTGTTTAGGTTC		
			FIP	CM1A2B FIPV2	GTGGGAAGGCTATGTCTGGCGGGGTTTTGGGAACTGAT		
			BIP	CM1A2B BIPV8	ATAAGCTTTTGACTCCTCCCTCCGGGCTAAAAGAAGAAGGATG		
		PCR	LP	CM01A	ACCCGGATCACTTCTTGGT	94 °C for 5 min; 35 cycles of 94 °C for 30 s, 64 °C for 30 s, and 72 °C for 1 min; 72 °C for 5 min	
			RP	CM02B	AAGAATGTTGTGTTTAGGTTC		
*Lamna nasus* (Porbeagle shark)	NADH2	LAMP	F3	LN02A	CCTGACAAAAACTTGCCCCT	65 °C for 60 min	444
			B3	LN07B	ATGGCTGGGATAACTAAGTTC		
			FIP	LN2A7B FIPV10	CTACTATGGTCGAGAGCACATTTACCCCTCATTAAATCCCAA		
			BIP	LN2A7B BIPV9	ACTTTCCATTATTGCCCTCATAACCAATAAGTCATTTTGGTATGAAGC		
			LF	LN2A7B F10B9 LFV1	CCAAGAAAGATAAGAAGG		
	COI	PCR	LP	LN06A	TGGCTCCCTCCTAGGCGAC	94 °C for 5 min; 35 cycles of 94 °C for 30 s, 66 °C for 30 s, and 72 °C for 1 min; 72 °C for 5 min	488
			RP	LN06B	GATCACACAAATAGGGGTGTC		
*Rhincodon typus* (Whale shark)	COI	LAMP	F3	RT02A	ATTACTTCCACCTTCATTCTTAT	70 °C for 50 min	265
			B3	RT04BI	TAGTTACAAGAATAGATCAGACG		
			FIP	RT2A4BI FIPV3	GATCAACTGATGCTCCCGCGCAGGAACAGGCTGAACTG		
			BIP	RT2A4BI BIPV7		CTCCTTACATCTAGCAGGAATTTCATGTTTGGTATTGAGAGATAGCT	
		PCR	LP	RT02A	ATTACTTCCACCTTCATTCTTAT	94 °C for 5 min; 35 cycles of 94 °C for 30 s, 62 °C for 30 s, and 72 °C for 1 min; 72 °C for 5 min	
			RP	RT04BI	TAGTTACAAGAATAGATCAGACG		
*Sphyrna lewini* (Scalloped hammerhead shark)	COI	LAMP	F3	SL04A	AGCTATCTTTTCTCTCCACC	68 °C for 60 min	174
			B3	SL04B	TGCAAGAACTGGAAGTGATAGG		
			FIP	SL4A4B FIPV1	AGGTTTCATGTTAATGATAGTAGCCGGTGTATCATCAATT		
			BIP	SL4A4B BIPV1	CCAGCCATTTCTCAATATCAAAGTAGGATAGTAGTTACAAGG		
		PCR	LP	SL04A	AGCTATCTTTTCTCTCCACC	94 °C for 5 min; 35 cycles of 94 °C for 30 s, 65 °C for 30 s, and 72 °C for 1 min; 72 °C for 5 min	
			RP	SL04B	TGCAAGAACTGGAAGTGATAGG		
*Sphyrna mokarran* (Great hammerhead shark)	COI	LAMP	F3	SM03A	CTTTCGTAATAATCTTTTTTATGGTA	70 °C for 50 min	389
			B3	SM04B	TAGTTACAAGAATAGATCAG		
			FIP	SM3A4B FIPV1	TTCGTGGGAAAGCTATATCTGGTGGGTGGTTTTGGGAATTGACT		
			BIP	SM3A4B BIPV1	CCACTTAGCTGGTATCTCATCAATCTTGAGAAATAGCTGGGGGT		
			LB	SM3A4B F1B1 LBV1	CTGGCCTCAATTAATTTCATCACAA		
		PCR	LP	SM03A	CTTTCGTAATAATCTTTTTTATGGTA	94 °C for 5 min; 35 cycles of 94 °C for 30 s, 59 °C for 30 s, and 72 °C for 1 min; 72 °C for 5 min	
			RP	SM04B	TAGTTACAAGAATAGATCAG		
*Sphyrna zygaena* (Smooth hammerhead shark)	COI	LAMP	F3	SZ03A	CATGAGCAGGTATAGTTGGG	68 °C for 60 min	299
			B3	SZ04B	TTCCTGCTCCAGCTTCTACC		
			FIP	SZ3A4B FIPV5	TGGTCATCTCCTAGAAGAGATCCAGCCCTAAGTCTCTTAATTCG		
			BIP	SZ3A4B BIPV2	AATCATAATTGGTGGCTTCGGGGGAGGAGAAGAAATGATGGTG		
		PCR	LP	SZ03A	CATGAGCAGGTATAGTTGGG	94 °C for 5 min; 35 cycles of 94 °C for 30 s, 68 °C for 30 s, and 72 °C for 1 min; 72 °C for 5 min	
			RP	SZ04B	TTCCTGCTCCAGCTTCTACC		
Internal control	12 S rRNA	LAMP	F3	UPCG01A	CCCCAAACTAGGATTAGATA	55 °C for 60 min	308
			B3	UPCG01B	ATGTAGCCCATTTCTATCCA		
			FIP	UPCG1A1B FIPV4	CTAGGTGGGTTTGGGACACCCGCCAGAGTACTACAAGC		
			BIP	UPCG1A1B BIPV7	TAAACCTCACCACTTCTGGCCTTTGCTACACCTCGACCT		
			LF	UPCG1A1B F4B7 LFV1	CCAAGTCCTTTGGGTTTYAAGCTAG		
			LB	UPCG1A1B F4B7 LBV3	CGTCGTCAGCYHACCYT		
	COI	PCR	LP	UP01A	YTTTGGTGCATGAGCAGG	94 °C for 5 min; 35 cycles of 94 °C for 30 s, 40 °C for 30 s, and 72 °C for 1 min; 72 °C for 5 min	148
			RP	UP01B	TAACTATAAAGAAGATTATTAC		

### Design of species-specific PCR primers and nucleic acid amplification assays using PCR techniques

Thirteen pairs (12 target shark species and one internal control) of PCR primers were designed using Primer-BLAST (https://www.ncbi.nlm.nih.gov/tools/primer-blast/). Species-specific PCR primers of bigeye thresher shark and oceanic whitetip shark were designed on the NADH2 region, whereas the primers for the rest were designed on the COI region. Sequences of primers and the corresponding PCR conditions are shown in Table 2. Species-specific PCR were tested against all collected non-target species using the GoTaq G2 Flexi DNA Polymerase (Promega, Wisconsin, USA) in T-100 Thermo Cycler (Bio-rad, California, USA) in triplicates. Each PCR mixture contained 10 ng/μL template DNA, 6 μL of 5X PCR buffer, 3 μL of MgCl_2_ (25 mmol/L), 0.6 μL of dNTP mixture (10 mmol/L each), 1.5 μL forward primer (10 μmol/L), 1.5 μL reverse primer (10 μmol/L) and 0.2 μL Taq polymerase (5 U/μL) with a final volume of 30 μL. PCR products were mixed with 6X loading dye in a ratio of 5:2 and visualized in 1.5% agarose gel. Sizes of fragments were compared with GeneRuler 100 bp DNA Ladder (Thermo Scientific, USA). Under this workflow, each PCR costed around USD $0.25.

### Sensitivity and specificity of the LAMP and PCR assays

The sensitivity of the LAMP and PCR assays was determined using genomic DNA of target species that was adjusted to a concentration of 10.0 ng/μL and diluted to 5.0 ng/μL, 1.0 ng/μL, 0.4 ng/μL, 0.2 ng/μL, and 0.1 ng/μL. The specificity of the LAMP and PCR assay was determined by amplification of the 93 shark and related species with the species-specific primers. All amplifications were performed in triplicates.

## Results

To evaluate the specificity of LAMP and PCR assays for the CITES-listed shark species, all species-specific primers (Table 2) were tested on their target species and against all non-target species as listed in Table 1. All samples were successfully amplified using universal COI primers^32,33^ and their identity confirmed using BLAST analysis. Most external primers (F3 and B3) of the LAMP primer sets were the same as their PCR forward and reverse primers (LP and RP) except for silky shark, porbeagle shark and internal control, as their LAMP and PCR primer sets were designed based on different regions. The expected size of target amplicon of each primer set is listed in Table 2.

### Species-specific LAMP assays

**Figure 1:**
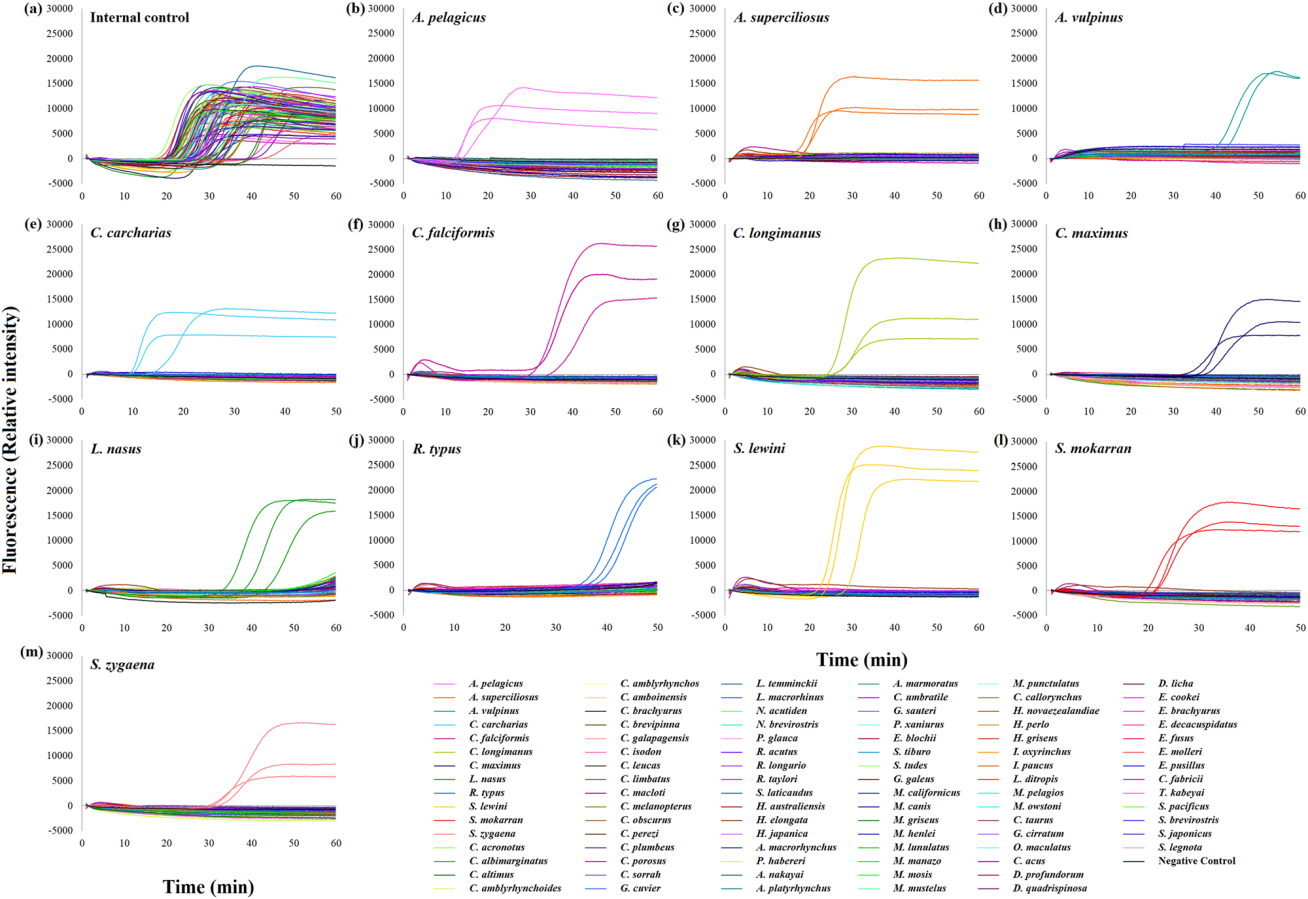
Amplification curves of LAMP assays for the CITES-listed shark species. Rising of fluorescence signals indicates positive amplification. (**a**) Amplification curves of all 93 species with specific primers for internal control. (**b–m**) Amplification results of all 93 species with species-specific primers targeting (**b**) pelagic thresher shark *Alopias pelagicus*, (**c**) bigeye thresher shark *Alopias superciliosus*, (**d**) common thresher shark *Alopias vulpinus*, (**e**) great white shark *Carcharodon carcharias*, (**f**) silky shark *Carcharhinus falciformis*, (**g**) oceanic whitetip shark *Carcharhinus longimanus*, (**h**) basking shark *Cetorhinus maximus*, (**i**) porbeagle shark *Lamna nasus*, (**j**) whale shark *Rhincodon typus*, (**k**) scalloped hammerhead shark *Sphyrna lewini*, (**l**) great hammerhead shark *Sphyrna mokarran*, and (**m**) smooth hammerhead shark *Sphyrna zygaena*.

**Figure 2:**
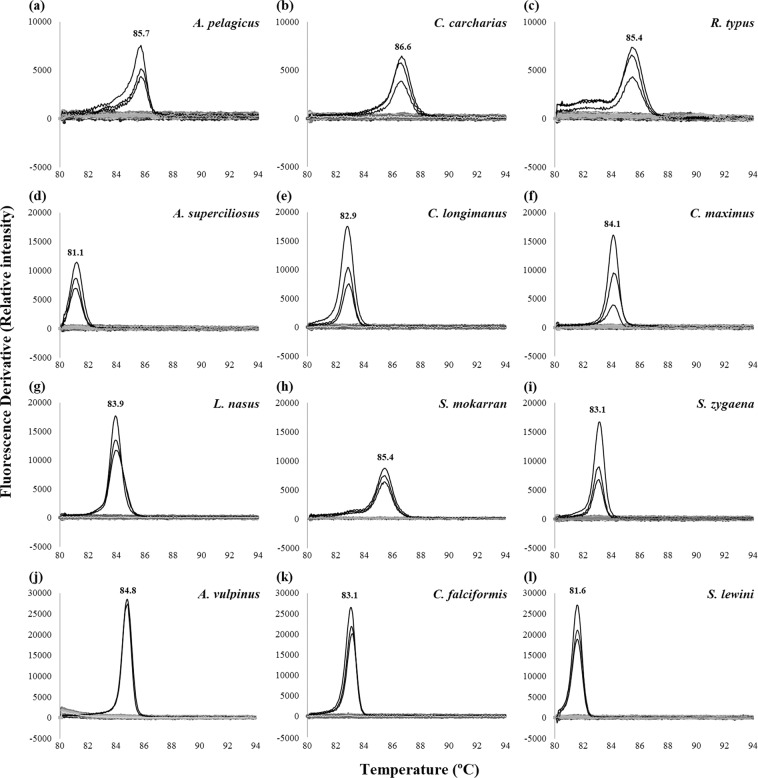
Melting curves of LAMP products for CITES-listed shark species. The melting temperature of the target species is shown as a peak. (**a–l**) Melting curves of products of LAMP assays targeting (**a**) pelagic thresher shark *Alopias pelagicus*, (**b**) great white shark *Carcharodon carcharias*, (**c**) whale shark *Rhincodon typus*, (**d**) bigeye thresher shark *Alopias superciliosus*, (**e**) oceanic whitetip shark *Carcharhinus longimanus*, (**f**) basking shark *Cetorhinus maximus*, (**g**) porbeagle shark *Lamna nasus*, (**h**) great hammerhead shark *Sphyrna mokarran*, (**i**) smooth hammerhead shark *Sphyrna zygaena*, (**j**) common thresher shark *Alopias vulpinus*, (**k**) silky shark *Carcharhinus falciformis*, and (**l**) scalloped hammerhead shark *Sphyrna lewini*.

**Figure 3:**
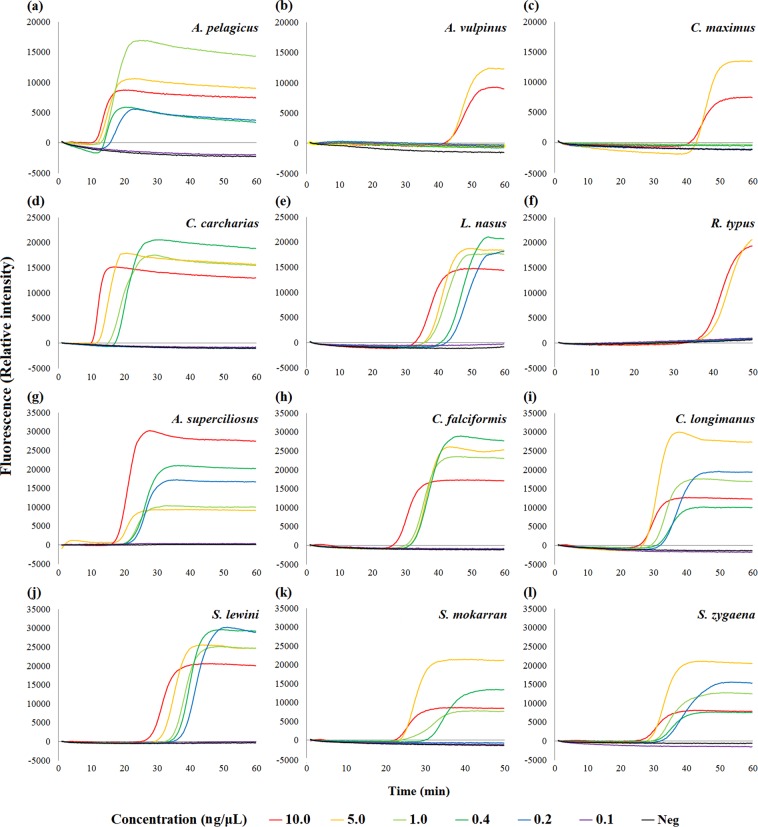
Sensitivity of LAMP assays for CITES-listed shark species. Sensitivity in terms of limit of detection of each LAMP assay for corresponding target species at 10.0 ng/µL, 5.0 ng/µL, 1.0 ng/µL, 0.4 ng/µL, 0.2 ng/µL, and 0.1 ng/µL. Rising of fluorescence signals indicates positive amplification. (**a–l**) Sensitivity of LAMP assays targeting (**a**) pelagic thresher shark *Alopias pelagicus*, (**b**) common thresher shark *Alopias vulpinus*, (**c**) basking shark *Cetorhinus maximus*, (**d**) great white shark *Carcharodon carcharias*, (**e**) porbeagle shark *Lamna nasus*, (**f**) whale shark *Rhincodon typus*, (**g**) bigeye thresher shark *Alopias superciliosus*, (**h**) silky shark *Carcharhinus falciformis*, (**i**) oceanic whitetip shark *Carcharhinus longimanus*, (**j**) scalloped hammerhead shark *Sphyrna lewini*, (**k**) great hammerhead shark *Sphyrna mokarran*, and (**l**) smooth hammerhead shark *Sphyrna zygaena*.

**Figure 4:**
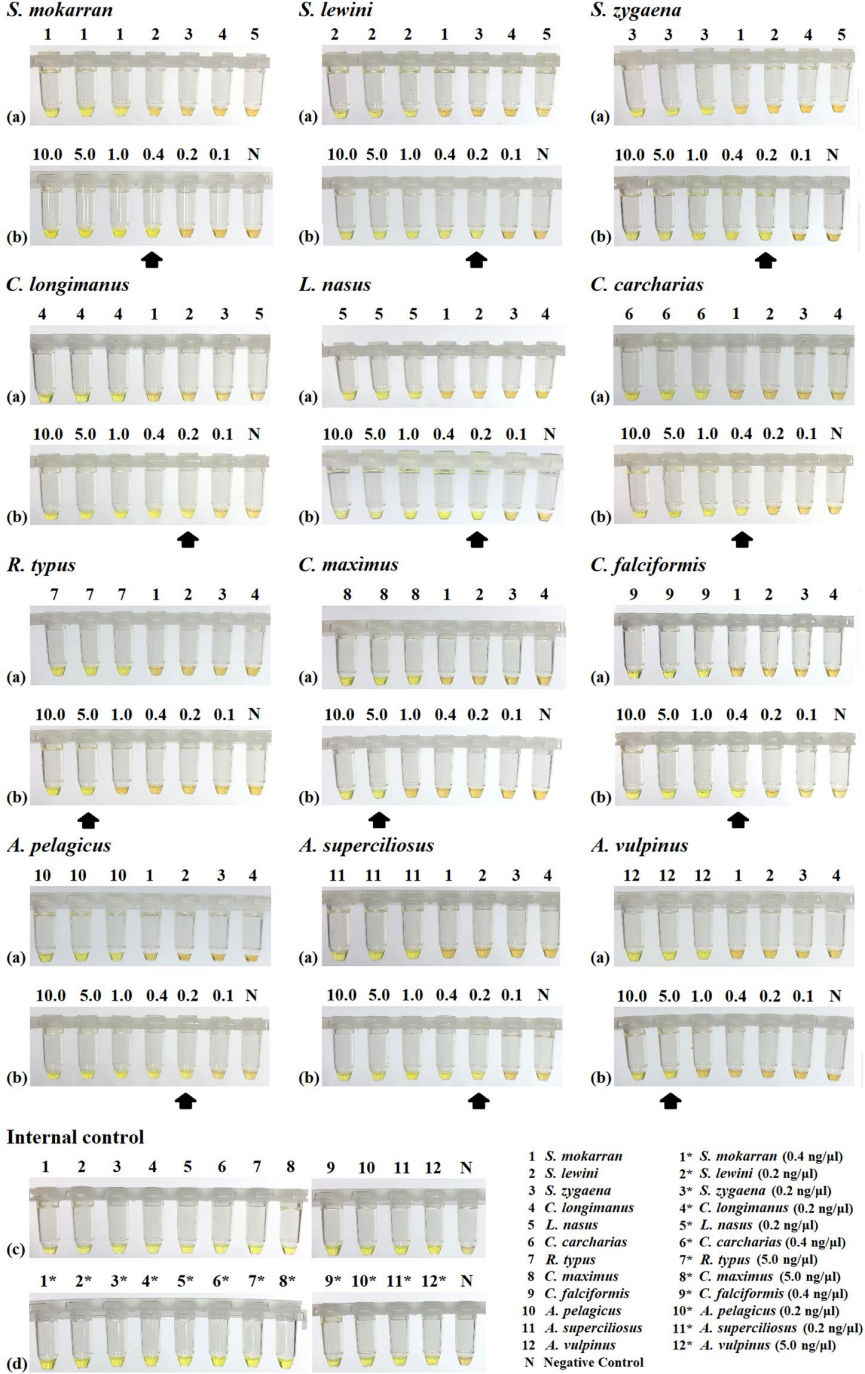
Visual detection of LAMP products of the CITES-listed shark species. End point assays were achieved by naked-eye visualization under ambient white light. Yellow colour represents positive LAMP results while brownish orange represents negative results. The upper panel, (**a,c**), shows results of target species against non-target species and negative control of each LAMP assay. The lower panel, (**b**) shows results of target species of the 12 species-specific LAMP assays at 10.0 ng/µL, 5.0 ng/µL, 1.0 ng/µL, 0.4 ng/µL, 0.2 ng/µL, and 0.1 ng/µL. Black arrow indicates the limit of detection of the corresponding species. For the lower panel of internal control, (**d**) shows the reactions with the 12 CITES-listed shark species at the concentration of their limit of detection in the species-specific LAMP assay.

The 12 CITES-listed shark species LAMP assays specifically amplified their target species only and non-target species were not detected (Fig. 1). Three independent experiments had been performed for each assay. The melting temperatures of LAMP products of the 12 LAMP assays were between 81.1 and 86.6 °C. The melting curves of target species with their corresponding LAMP assays were identical and no melting curve was found for non-target species showing the specificity of these LAMP assays (Fig. 2). For the sensitivity, the detection limit of the LAMP assay was 0.2 ng/μL for pelagic thresher shark, bigeye thresher shark, oceanic whitetip shark, porbeagle shark, scalloped hammerhead shark and smooth hammerhead shark; 0.4 ng/μL for great white shark, silky shark, and great hammerhead shark and 5.0 ng/μL for common thresher shark, basking shark and whale shark (Fig. 3). For the internal control of the LAMP assay, positive result was found with all samples and no amplification was found with the negative control. The LAMP assay for internal control was able to detect the 12 CITES-listed shark species at the concentration equivalent to the detection limit of their corresponding species-specific LAMP assays (Supporting Information). Apart from the real-time amplification curve and melting curve obtained from Genie II, positive LAMP signals could be observed by naked eye after addition of SYBR Green I dye. As shown in Fig. 4, under ambient white light, all positive LAMP reactions turned to yellow colour and negative reactions remained in brownish orange colour (Fig. 4a,c). The lower panel (Fig. 4b) shows the limit of detection of each LAMP assay under visual detection. These features allowed rapid on-site screening.

### Species-specific PCR assays

All CITES-listed shark species were specifically amplified by their corresponding PCR assays with expected sizes (Table 2) and no amplification was found for non-target species, showing the specificity of each of the PCR assays (Supporting Information). The limit of detection of each PCR assay was 0.2 ng/μL for porbeagle shark and bigeye thresher shark; 0.4 ng/μL for common thresher shark, oceanic whitetip shark, and smooth hammerhead shark; 5.0 ng/μL for pelagic thresher shark, great white shark, silky shark, basking shark, scalloped hammerhead shark and great hammerhead shark; 10.0 ng/μL for whale shark (Supporting Information). All samples showed positive results in the PCR assay for internal control and no amplification was found in the negative control. Internal control could be successfully amplified from the 12 CITES-listed shark species at the DNA concentration equivalent to the limit of detection of their corresponding species-specific PCR assay (Supporting Information).

## Discussion

In this work, we have developed sensitive, simple to use, and to our knowledge, the fastest species-specific assays using LAMP technique, as well as simple and low-cost PCR assays to authenticate all 12 CITES-listed shark species. The LAMP and PCR assays we developed can successfully discriminate their target species from the other 92 shark and related species, which has included species commonly found in the Hong Kong shark fin market^12^.

We have designed our primers based on COI and NADH2 loci for the CITES-listed shark species and on 12 s rRNA locus for internal control. Previous studies about species-specific primers on shark species identification are mostly focused on the ITS2 locus^17–20^. The COI locus, a standard marker for fish and shark species DNA barcoding^16,32,33,35,36^, is highly species specific and the number of nucleotide difference of congeneric species is sufficient for designing species-specific primers. For common thresher shark, silky shark, oceanic whitetip shark and porbeagle shark, NADH2 locus is a better region for designing species-specific primer, due to high nucleotide sequence differences between the target species and the corresponding congeneric species^37^. High degree of nucleotide sequence difference allows us to design LAMP primers targeting four to six species-specific regions instead of only two for conventional PCR. Our LAMP assays are highly specific, especially for species with many congeneric species such as the silky shark and the ocean whitetip shark. Furthermore, care was taken to avoid sites of known intraspecific variation among sharks from different regions or ocean basins during primer design, so that amplification success would not be affected by intraspecific nucleotide variation. In contrast, 12 s rRNA locus is a suitable target for designing primers for the internal control as it has high sequence homology among different shark species. This allows us to design six LAMP primers that can amplify all of the shark species in this work.

Samples of 91 shark species and 2 Chimaera species were collected for testing the specificity of our developed LAMP and PCR assays. These species covered 56 out of 61 species found in the Hong Kong fin market in 2014–2015^12^ The other five species were rarely found in the fin trade market. For LAMP and PCR assays targeting the silky shark and the oceanic whitetip shark, we have tested 21 out of 31 congeneric species from *Carcharhinus* and a total of 76 *Carcharhinus* samples were tested. Although we were not able to test the assays on all 31 recognised *Carcharhinus* species^37^, we have confidence on the specificity of our assays for three reasons. First, we have tested our assays against most species which are phylogenetically close to the silky shark and the oceanic whitetip shark^38,39^ and no amplification was found with non-target species. Second, those nine untested species are phylogenetically more distant from the silky shark and the oceanic whitetip shark^39^ and they are rarely found in the Hong Kong fin market^12^. Third, we have compared nucleotide sequences of those nine species on NADH2 region available on NCBI GenBank except for *C. hemiodon*, which has no sequences available on any public database, for checking of primer mismatching with Primer-BLAST to ensure the specificity of our primers. For the oceanic whitetip shark, previous studies have found difficulties on designing species-specific primers targeting the oceanic whitetip shark due to its high phylogenetically similarity with *C. obscurus* and *C. galapagensis*^17,22,39^. We are the first to successfully develop species-specific assays for the oceanic whitetip shark. We have found that these three species share less similarity among their sequences on the NADH2 region and hence easier to design species-specific primers discriminating the oceanic whitetip shark from the other two species. Furthermore, we have used DNA extracted from processed shark fin for testing to ensure that the LAMP assays could provide accurate result when performed on-field.

Besides oceanic whitetip shark, we are also the first to present species-specific assays for the whale shark. Whale shark, being the only species in the family Rhincodontidae, is phylogenetically more distant from other species in Orectolobiformes^37,39,40^. We have examined the COI sequences in Orectolobiformes that are available on the NCBI database and performed the Primer-BLAST analysis to ensure the specificity of our assays. For the thresher sharks, basking shark, great white shark and porbeagle shark, they are classified in the mackerel shark order Lamniformes. For the LAMP and PCR assays targeting these six species, we have tested them with 12 out of 15 recognised species of the order Lamniformes including all species in the family Alopiidae and the family Lamnidae to ensure the specificity of our primers. For the three CITES-listed hammerhead sharks, we have tested our assays with six out of nine species in the family Sphyrnidae. The other three species (*S. corona*, *S. gilberti*, and *S. media*) are phylogenetically more distant from the CITES-listed hammerhead sharks and not commonly found in the Hong Kong shark fin market^3,12,19,41,42^.

LAMP allows rapid amplification of DNA using *Bst* polymerase with high strand displacement activity at isothermal temperature within an hour. This facilitates diagnostic on-site detection with limited equipment and provides species-level identification in a short period of time, increasing efficiency for law enforcement. To our knowledge, this is the first report on applying LAMP on shark species identification. Although the optimal temperature for *Bst* polymerase is between 60 °C and 65 °C^43^, the optimal reaction temperatures for most of the LAMP assays developed were found to be 68 °C or 70 °C, except for those targeting bigeye thresher shark, common thresher shark and porbeagle shark. Increase in LAMP reaction temperature allows a better specificity especially for eliminating congeneric species. Apart from increasing reaction temperature, another common approach of optimizing LAMP reaction is the addition of loop primers, which can increase the sensitivity of LAMP reaction. The LAMP assays targeting great white shark, porbeagle shark, great hammerhead shark, and that for the internal control include loop primers in their primer sets. Loop primers are designed to bind to the additional sites that are not accessed by the internal primers and accelerate the rate of LAMP reaction and provide higher sensitivity^24^. This allows the LAMP assay for the internal control to amplify all shark species in Table 1 at a relatively low reaction temperature, which also favours primer annealing and amplification. The presence of loop primers also allows the LAMP assays developed to give amplification with detectable threshold within 60 minutes, reducing the reaction time required for the assays. For LAMP and PCR assays amplifying the same region of a species, the species-specific LAMP assays generally have a better sensitivity than the species-specific PCR assays, except for the LAMP assays targeting common thresher shark and basking shark. Looped DNA product increases the sensitivity of the LAMP assay and hence facilitates detection with limited sample amount from dried shark fins. In addition to better sensitivity, since LAMP produces more DNA products than conventional PCR method, it allows easy visual detection with naked eye to tell between positive and negative results without fluorescence detection equipment. On-site detection of CITES-listed shark species can be achieved. On the other hand, there are also limitations on LAMP assays. Since LAMP assay is highly sensitive, it is important to perform them at their optimal reaction temperature. Four different temperatures, according to conditions listed on Table 2 should be used to achieve repeatable result. Furthermore, there may be a chance of cross contamination leading to false positive results. Therefore, it is important to clean the equipment before test and keep good laboratory practice. Since there are only 16 wells available on Genie II, using of multi-block thermal cycler may be considered.

For on-field screening and monitoring of shark fin products with unknown identity, use of multi-block thermal cycler is suggested for performing LAMP reactions, at four different temperatures within 60 minutes. Sample DNA, in different forms including dried processed shark fin, dried and frozen flesh tissues and inner organs, can be extracted using (1) Biomed Genomic DNA/Tissue Extraction Kit in 30–60 minutes depending on the rate of sample dissolution in lysis buffer; or (2) Kaneka Easy DNA Extraction Kit (Version 2) (Funakoshi, Osaka, Japan) in 15 minutes. After sample extraction, 1 μL of the extracted DNA is amplified by LAMP (50–60 minutes). At the end of the reaction, 2 μL of 1000X SYBR Green dye is added for visual detection. If there is positive LAMP reaction, the colour would change simultaneously with the addition of SYBR Green dye. For a 96-well multi-block thermal cycler with temperature control in each column, eight samples, including positive and negative control, with 12 LAMP assays could be tested per hour. To obtain the best result, portable device for DNA concentration measurement could also be used. In our experience, DNA with concentration of at least 10.0 ng/µL was able to be extracted from all unprocessed and processed samples using the Biomed Genomic DNA/Tissue Extraction Kit.

There are several on-field detection protocols available for CITES-listed shark species identification. They include multiplex real-time PCR assay by Cardeñosa *et al*.^22^ and genome skimming using MinION hand-held sequencer by Johri *et al*.^44^. Our protocol has good potential to be used on field. As shown in Table 3, our LAMP assays have covered all twelve CITES-listed shark species and it is cheaper in the aspects of cost and equipment compared to the other two methods. It is less time consuming for test on a small scale and allows simple result analysis which is more favourable to customs officials without scientific background. Multiplex real-time PCR assay allows test on 94 samples within 4 hours, which is the fastest one among these three methods and data analysis is also straightforward. This could be favourable for on-field test on a large scale. However, the multiplex real-time PCR developed only covers 9 out of 12 CITES-listed shark species. For the other three shark species, use of morphological guide is needed^22^. For genome skimming using MinION hand-held sequencer, the major limiting factors are the high cost of MinION flow cell compared to the other two methods. Although this protocol could provide additional genetic information, result has to be analysed by bioinformatic expertise, which is less favourable for customs use. Furthermore, this approach has not been tested with highly processed shark fin products which are generally skinned and bleached. In short, for on-field detection by customs, LAMP assays are more favourable for small-scaled quick test while multiplex real-time PCR assay is more favourable for large-scaled test.

**Table 3:** Comparison of on-field detection protocols for CITES-listed shark species identification.

		LAMP assay	Multiplex real-time PCR assay^a^	Genome skimming using MinION hand-held sequencer^b^
Cost per sample		USD$ 0.6	USD$ 0.94	USD$ 0.8 (Extraction only)
Equipment		Multi-block thermal cycler	Real-time thermal cycler	MinION sequencer
Time required	Number of sample			
	1	3 hours	3 hours	12–40 hours
	10	3 hours	3 hours	12–40 hours
	94	14 hours	4 hours	12–40 hours
Tested with processed shark fin		Yes	Yes	No
Bioinformatics		Simple	Simple	Need expertise
Coverage of CITES species		All 12 species	9 out of 12 species	All 12 species
Additional genetic information		No	No	Yes

^a^Cardeñosa *et al*.^22^.

^b^Johri *et al*.^44^.

Our rapid on-site detection assays have shown good specificity and can be used for on-site law enforcement of all CITES-listed shark species which are commonly found in the fin market^3,12^. It will be useful for screening of processed shark fin products which is difficult to be identified using morphological identification guide and shark products such as shark meat as they are hardly identified based on morphology^22,45^. The result has also shown that COI and NADH2 regions are suitable for species-level identification, especially for species with a high nucleotide similarity with congeneric species.

Our LAMP assays allow easy inspection of imported shark fin products at the border for all 12 CITES-listed shark species and facilitate monitoring of international trade^46^. Same model using our LAMP assays could also be applied for law enforcement in other area. For example, as the 4th largest shark-catching area^47^, Taiwan has recently passed the “Regulations for Import of Shark Fins” of which catching of whale shark and import of whale shark products are forbidden^48^. Our protocol for whale shark could provide a reliable and efficient test for monitoring the trade of whale shark products. With the exact species identity of shark fin products, it would provide a better picture on the CITES-listed shark products in Hong Kong and elsewhere. The data could be important for better monitoring of international shark fin trade and the conservation of shark by CITES members^49^.

Besides shark species currently listed on CITES appendix II, there is a need for development of rapid identification assay for other endangered shark species in the future CITES appendix and the list of IUCN to ensure effective detection of illegal trade. In fact, two more shark species, *Isurus oxyrinchus* and *I. paucus*, have been recently included in CITES Appendix II at the 18th meeting of the Conference of the Parties of the CITES in 2019 and will enter into effect on 26 November 2019^11^. Our innovation can be extended to these new CITES-listed shark species and processed products and for further development of multiplex LAMP assays. We also expect that our work will serve as a model for the rapid identification assay for other endangered species.

## Supplementary information

Supplementary Information.
