# Supplementary material for: Rapid detection of CITES-listed shark fin species by loop-mediated isothermal amplification assay with potential for field use
Source: Sci Rep. 2020 Mar 10;10:4455. doi: 10.1038/s41598-020-61150-8 (PMC7064571; doi:10.1038/s41598-020-61150-8)

Rapid detection of CITES-listed shark fin species by loop-mediated isothermal amplification assay with potential for field use

Grace Wing-Chiu BUT[1], Hoi-Yan WU[2,3], Kwang-Tsao SHAO[4], Pang-Chui SHAW[1,2,3*]

[1] School of Life Sciences, The Chinese University of Hong Kong, Hong Kong

[2] Institute of Chinese Medicine, The Chinese University of Hong Kong, Hong Kong

[3] Li Dak Sum Yip Yio Chin R & D Centre for Chinese Medicine, The Chinese University of Hong Kong, Hong Kong

[4] Systematics and Biodiversity Information Division, Biodiversity Research Center, Academia Sinica, Taiwan

- Corresponding authors: Pang-Chui Shaw; School of Life Sciences, The Chinese University of Hong Kong, Hong Kong; Tel: +852-3943-1363; Fax: +852-2603-7246; Email: pcshaw@cuhk.edu.hk

Supplementary Material

Appendix S1 *Amplification curves of LAMP assay for internal control; results of amplification reactions and sensitivity of PCR assays for CITES-listed shark species and internal control*

Figure S1 | The upper panel, **(a)**, is the results of amplification reactions of the 12 CITES-listed shark species with the specific primers for internal control at the limit of detection of their corresponding species-specific PCR assays. The lower panel, **(b)**, amplification curves of the 12 CITES-listed shark species with the specific primers for internal control at the limit of detection of their corresponding species-specific LAMP assays. Rising of fluorescence signals indicates positive amplification. Clear amplification were achieved with both PCR and LAMP assays for internal control.

Figure S2 | Amplification results of all 93 species with specific PCR primers for internal control.

Figure S3 | Amplification results of all 93 species with species-specific PCR primers targeting pelagic thresher shark *Alopias pelagicus.*

Figure S4 | Amplification results of all 93 species with species-specific PCR primers targeting bigeye thresher shark *Alopias superciliosus.*

Figure S5 | Amplification results of all 93 species with species-specific PCR primers targeting common thresher shark *Alopias vulpinus.*

Figure S6 | Amplification results of all 93 species with species-specific PCR primers targeting great white shark *Carcharodon carcharias.*

Figure S7 | Amplification results of all 93 species with species-specific PCR primers targeting silky shark *Carcharhinus falciformis.*

Figure S8 | Amplification results of all 93 species with species-specific PCR primers targeting oceanic whitetip shark *Carcharhinus longimanus.*

Figure S9 | Amplification results of all 93 species with species-specific PCR primers targeting basking shark *Cetorhinus maximus.*

Figure S10 | Amplification results of all 93 species with species-specific PCR primers targeting porbeagle shark *Lamna nasus*.

Figure S11 | Amplification results of all 93 species with species-specific PCR primers targeting whale shark *Rhincodon typus*.

Figure S12 | Amplification results of all 93 species with species-specific PCR primers targeting scalloped hammerhead shark *Sphyrna lewini.*

Figure S13 | Amplification results of all 93 species with species-specific PCR primers targeting great hammerhead shark *Sphyrna mokarran.*

Figure S14 | Amplification results of all 93 species with species-specific PCR primers targeting smooth hammerhead shark *Sphyrna zygaena*.

Figure S15 | Sensitivity of the CITES-listed shark species PCR assay targeting pelagic thresher shark *Alopias pelagicus* with corresponding target species at the concentration of: lane 1, 10.0 ng/µL; 2, 5.0 ng/µL; 3, 1.0 ng/µL; 4, 0.4 ng/µL; 5, 0.2 ng/µL;6, 0.1 ng/µL; N, negative control. Sensitivity in terms of limit of detection of each PCR assay for corresponding target species with different concentrations, 10.0 ng/µL, 5.0 ng/µL, 1.0 ng/µL, 0.4 ng/µL, 0.2 ng/µL, and 0.1 ng/µL.

Figure S16 | Sensitivity of the CITES-listed shark species PCR assay targeting bigeye thresher shark *Alopias superciliosus* with corresponding target species at the concentration of: lane 1, 10.0 ng/µL; 2, 5.0 ng/µL; 3, 1.0 ng/µL; 4, 0.4 ng/µL; 5, 0.2 ng/µL;6, 0.1 ng/µL; N, negative control. Sensitivity in terms of limit of detection of each PCR assay for corresponding target species with different concentrations, 10.0 ng/µL, 5.0 ng/µL, 1.0 ng/µL, 0.4 ng/µL, 0.2 ng/µL, and 0.1 ng/µL.

Figure S17 | Sensitivity of the CITES-listed shark species PCR assay targeting common thresher shark *Alopias vulpinus* with corresponding target species at the concentration of: lane 1, 10.0 ng/µL; 2, 5.0 ng/µL; 3, 1.0 ng/µL; 4, 0.4 ng/µL; 5, 0.2 ng/µL;6, 0.1 ng/µL; N, negative control. Sensitivity in terms of limit of detection of each PCR assay for corresponding target species with different concentrations, 10.0 ng/µL, 5.0 ng/µL, 1.0 ng/µL, 0.4 ng/µL, 0.2 ng/µL, and 0.1 ng/µL.

Figure S18 | Sensitivity of the CITES-listed shark species PCR assay targeting great white shark *Carcharodon carcharias* with corresponding target species at the concentration of: lane 1, 10.0 ng/µL; 2, 5.0 ng/µL; 3, 1.0 ng/µL; 4, 0.4 ng/µL; 5, 0.2 ng/µL;6, 0.1 ng/µL; N, negative control. Sensitivity in terms of limit of detection of each PCR assay for corresponding target species with different concentrations, 10.0 ng/µL, 5.0 ng/µL, 1.0 ng/µL, 0.4 ng/µL, 0.2 ng/µL, and 0.1 ng/µL.

Figure S19 | Sensitivity of the CITES-listed shark species PCR assay targeting silky shark *Carcharhinus falciformis* with corresponding target species at the concentration of: lane 1, 10.0 ng/µL; 2, 5.0 ng/µL; 3, 1.0 ng/µL; 4, 0.4 ng/µL; 5, 0.2 ng/µL;6, 0.1 ng/µL; N, negative control. Sensitivity in terms of limit of detection of each PCR assay for corresponding target species with different concentrations, 10.0 ng/µL, 5.0 ng/µL, 1.0 ng/µL, 0.4 ng/µL, 0.2 ng/µL, and 0.1 ng/µL.

Figure S20 | Sensitivity of the CITES-listed shark species PCR assay targeting oceanic whitetip shark *Carcharhinus longimanus* with corresponding target species at the concentration of: lane 1, 10.0 ng/µL; 2, 5.0 ng/µL; 3, 1.0 ng/µL; 4, 0.4 ng/µL; 5, 0.2 ng/µL;6, 0.1 ng/µL; N, negative control. Sensitivity in terms of limit of detection of each PCR assay for corresponding target species with different concentrations, 10.0 ng/µL, 5.0 ng/µL, 1.0 ng/µL, 0.4 ng/µL, 0.2 ng/µL, and 0.1 ng/µL.

Figure S21 | Sensitivity of the CITES-listed shark species PCR assay targeting basking shark *Cetorhinus maximus* with corresponding target species at the concentration of: lane 1, 10.0 ng/µL; 2, 5.0 ng/µL; 3, 1.0 ng/µL; 4, 0.4 ng/µL; 5, 0.2 ng/µL;6, 0.1 ng/µL; N, negative control. Sensitivity in terms of limit of detection of each PCR assay for corresponding target species with different concentrations, 10.0 ng/µL, 5.0 ng/µL, 1.0 ng/µL, 0.4 ng/µL, 0.2 ng/µL, and 0.1 ng/µL.

Figure S22 | Sensitivity of the CITES-listed shark species PCR assay targeting porbeagle shark *Lamna nasus* with corresponding target species at the concentration of: lane 1, 10.0 ng/µL; 2, 5.0 ng/µL; 3, 1.0 ng/µL; 4, 0.4 ng/µL; 5, 0.2 ng/µL;6, 0.1 ng/µL; N, negative control. Sensitivity in terms of limit of detection of each PCR assay for corresponding target species with different concentrations, 10.0 ng/µL, 5.0 ng/µL, 1.0 ng/µL, 0.4 ng/µL, 0.2 ng/µL, and 0.1 ng/µL.

Figure S23 | Sensitivity of the CITES-listed shark species PCR assay targeting whale shark *Rhincodon typus* with corresponding target species at the concentration of: lane 1, 10.0 ng/µL; 2, 5.0 ng/µL; 3, 1.0 ng/µL; 4, 0.4 ng/µL; 5, 0.2 ng/µL;6, 0.1 ng/µL; N, negative control. Sensitivity in terms of limit of detection of each PCR assay for corresponding target species with different concentrations, 10.0 ng/µL, 5.0 ng/µL, 1.0 ng/µL, 0.4 ng/µL, 0.2 ng/µL, and 0.1 ng/µL.

Figure S24 | Sensitivity of the CITES-listed shark species PCR assay targeting scalloped hammerhead shark *Sphyrna lewini* with corresponding target species at the concentration of: lane 1, 10.0 ng/µL; 2, 5.0 ng/µL; 3, 1.0 ng/µL; 4, 0.4 ng/µL; 5, 0.2 ng/µL;6, 0.1 ng/µL; N, negative control. Sensitivity in terms of limit of detection of each PCR assay for corresponding target species with different concentrations, 10.0 ng/µL, 5.0 ng/µL, 1.0 ng/µL, 0.4 ng/µL, 0.2 ng/µL, and 0.1 ng/µL.

Figure S25 | Sensitivity of the CITES-listed shark species PCR assay targeting great hammerhead shark *Sphyrna mokarran* with corresponding target species at the concentration of: lane 1, 10.0 ng/µL; 2, 5.0 ng/µL; 3, 1.0 ng/µL; 4, 0.4 ng/µL; 5, 0.2 ng/µL;6, 0.1 ng/µL; N, negative control. Sensitivity in terms of limit of detection of each PCR assay for corresponding target species with different concentrations, 10.0 ng/µL, 5.0 ng/µL, 1.0 ng/µL, 0.4 ng/µL, 0.2 ng/µL, and 0.1 ng/µL.

Figure S26 | Sensitivity of the CITES-listed shark species PCR assay targeting smooth hammerhead shark *Sphyrna zygaena* with corresponding target species at the concentration of: lane 1, 10.0 ng/µL; 2, 5.0 ng/µL; 3, 1.0 ng/µL; 4, 0.4 ng/µL; 5, 0.2 ng/µL;6, 0.1 ng/µL; N, negative control. Sensitivity in terms of limit of detection of each PCR assay for corresponding target species with different concentrations, 10.0 ng/µL, 5.0 ng/µL, 1.0 ng/µL, 0.4 ng/µL, 0.2 ng/µL, and 0.1 ng/µL.

Figure S1 | The upper panel, **(a)**, is the results of amplification reactions of the 12 CITES-listed shark species with the specific primers for internal control at the limit of detection of their corresponding species-specific PCR assays. The lower panel, **(b)**, amplification curves of the 12 CITES-listed shark species with the specific primers for internal control at the limit of detection of their corresponding species-specific LAMP assays. Rising of fluorescence signals indicates positive amplification. Clear amplification were achieved with both PCR and LAMP assays for internal control.

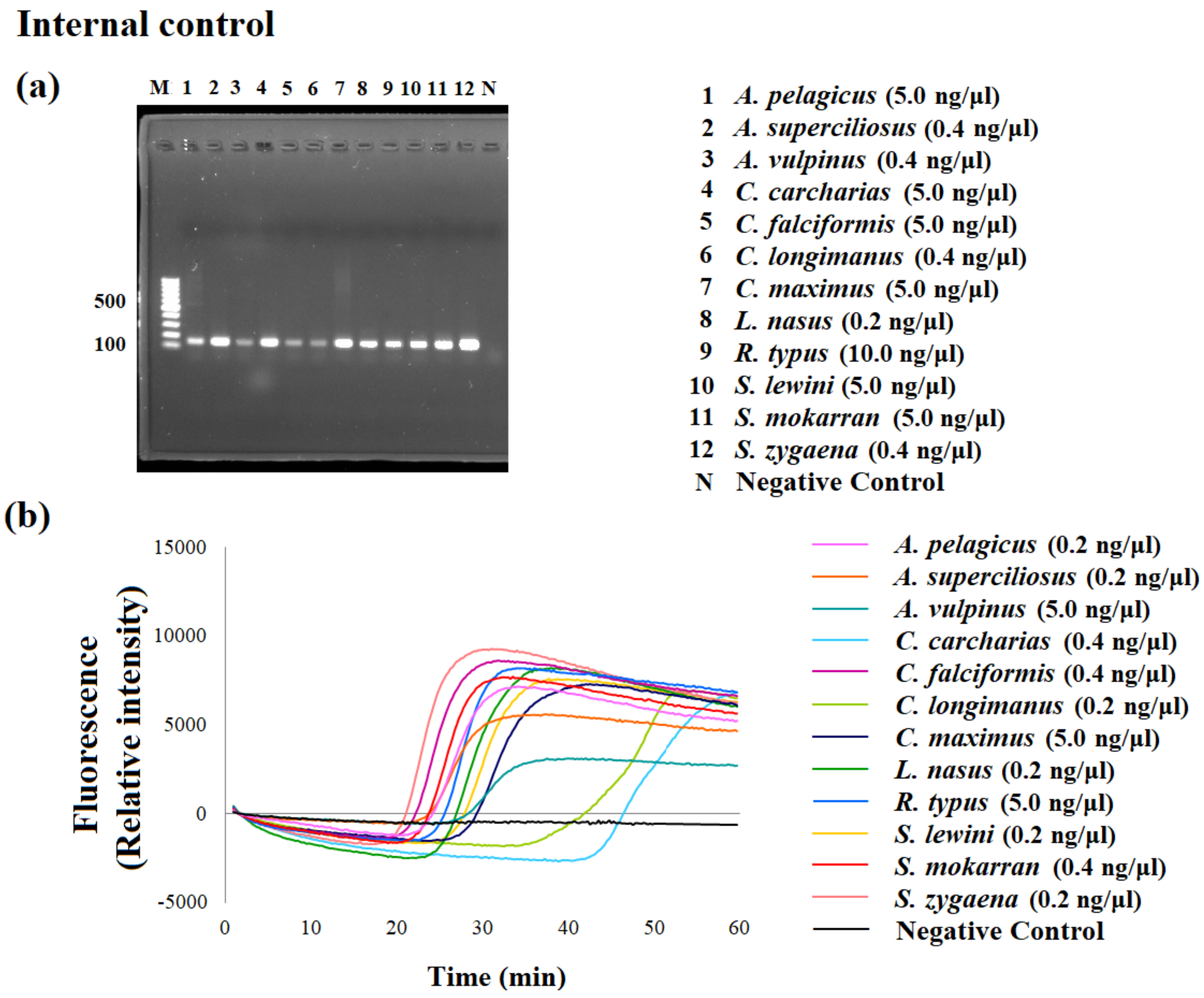

Figure S2 Amplification results of all 93 species with specific PCR primers for internal control.

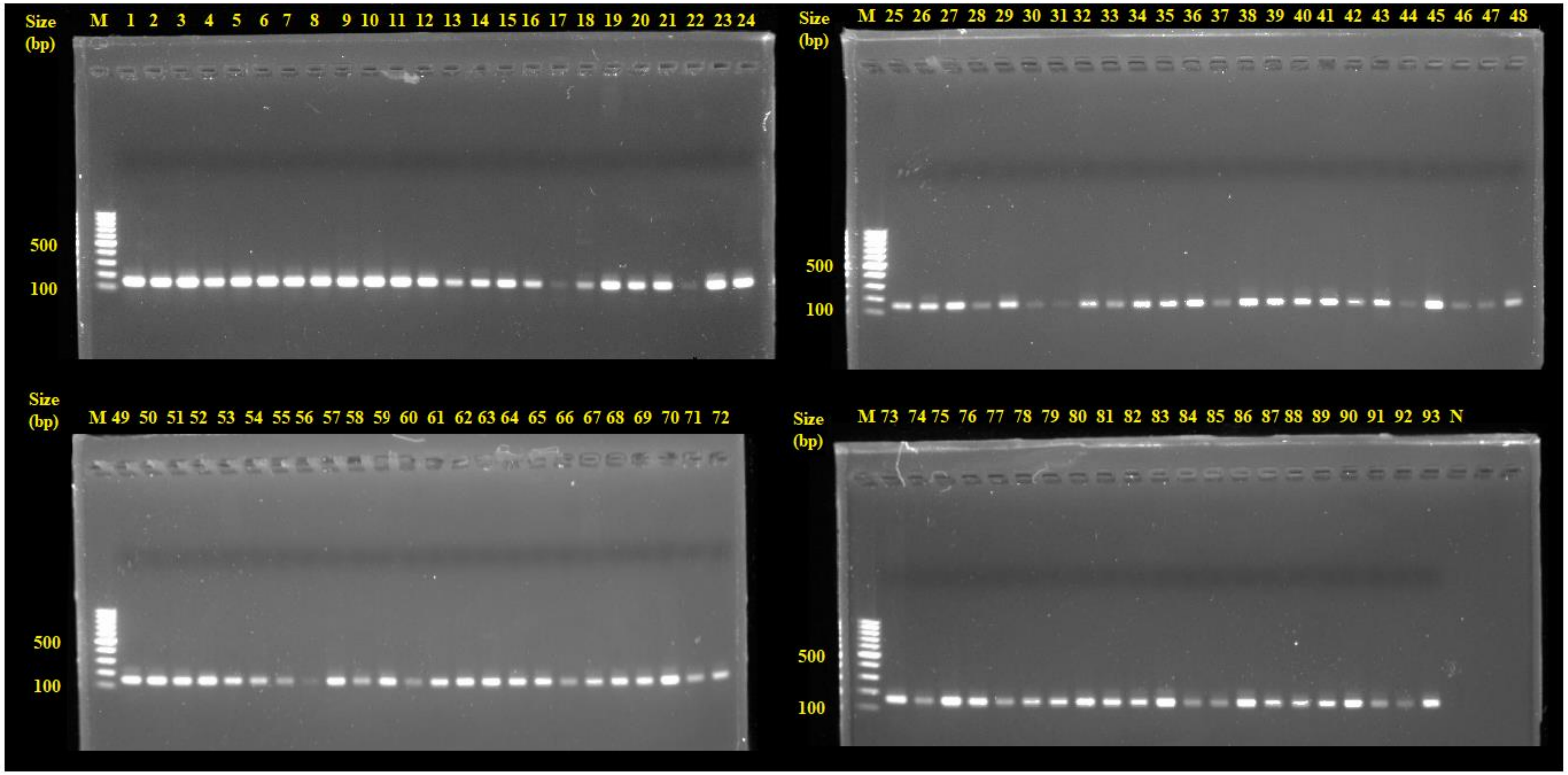

1 *S. mokarran*
2 *S. lewini*
3 *S. zygaena*
4 *C. longimanus*
5 *L. nasus*
6 *C. carcharias*
7 *R. typus*
8 *C. maximus*
9 *C. falciformis*
10 *A. pelagicus*
11 *A. superciliosus*
12 *A. vulpinus*
13 *A. macrorhynchus*
14 *A. nakayai*
15 *A. platyrhynchus*
16 *A. marmoratus*
17 *C. albimarginatus*
18 *C. altimus*
19 *C. brachyurus*
20 *C. brevipinna*
21 *C. leucas*
22 *C. limbatus*
23 *C. melanopterus*
24 *C. obscurus*
25 *C. perezi*
26 *C. plumbeus*
27 *C. sorrah*
28 *C. acus*
29 *C. fabricii*
30 *C. umbratile*
31 *D. quadrispinosa*
32 *E. cookei*
33 *E. brachyurus*
34 *E. decacuspidatus*
35 *E. fusus*
36 *E. molleri*
37 *E. pusillus*
38 *G. cuvier*
39 *G. sauteri*
40 *G. cirratum*
41 *H. japanica*
42 *H. perlo*
43 *I. oxyrinchus*
44 *I. paucus*
45 *L. ditropis*
46 *M. pelagios*
47 *M. owstoni*
48 *M. griseus*
49 *M. manazo*
50 *N. acutiden*
51 *N. brevirostris*
52 *O. maculatus*
53 *P. xaniurus*
54 *P. glauca*
55 *P. habereri*
56 *S. pacificus*
57 *S. tudes*
58 *S. brevirostris*
59 *S. japonicus*
60 *S. legnota*
61 *T. kabeyai*
62 *C. callorynchus*
63 *C. acronotus*
64 *C. amblyrhynchoides*
65 *C. amblyrhynchos*
66 *C. amboinensis*
67 *C. galapagensis*
68 *C. isodon*
69 *C. macloti*
70 *C. porosus*
71 *Carcharias taurus*
72 *Dalatias licha*
73 *D. profundorum*
74 *E. blochii*
75 *G. galeus*
76 *H. australiensis*
77 *H. elongata*
78 *H. griseus*
79 *H. novaezealandiae*
80 *L. temminckii*
81 *L. macrorhinus*
82 *M. californicus*
83 *M. canis*
84 *M. henlei*
85 *M. lunulatus*
86 *M. mosis*
87 *M. mustelus*
88 *M. punctulatus*
89 *R. acutus*
90 *R. longurio*
91 *R. taylori*
92 *S. laticaudus*
93 *S. tiburo*
N Negative Control
M 100 bp DNA marker
E Empty

Figure S3 Amplification results of all 93 species with species-specific PCR primers targeting pelagic thresher shark *Alopias pelagicus*.

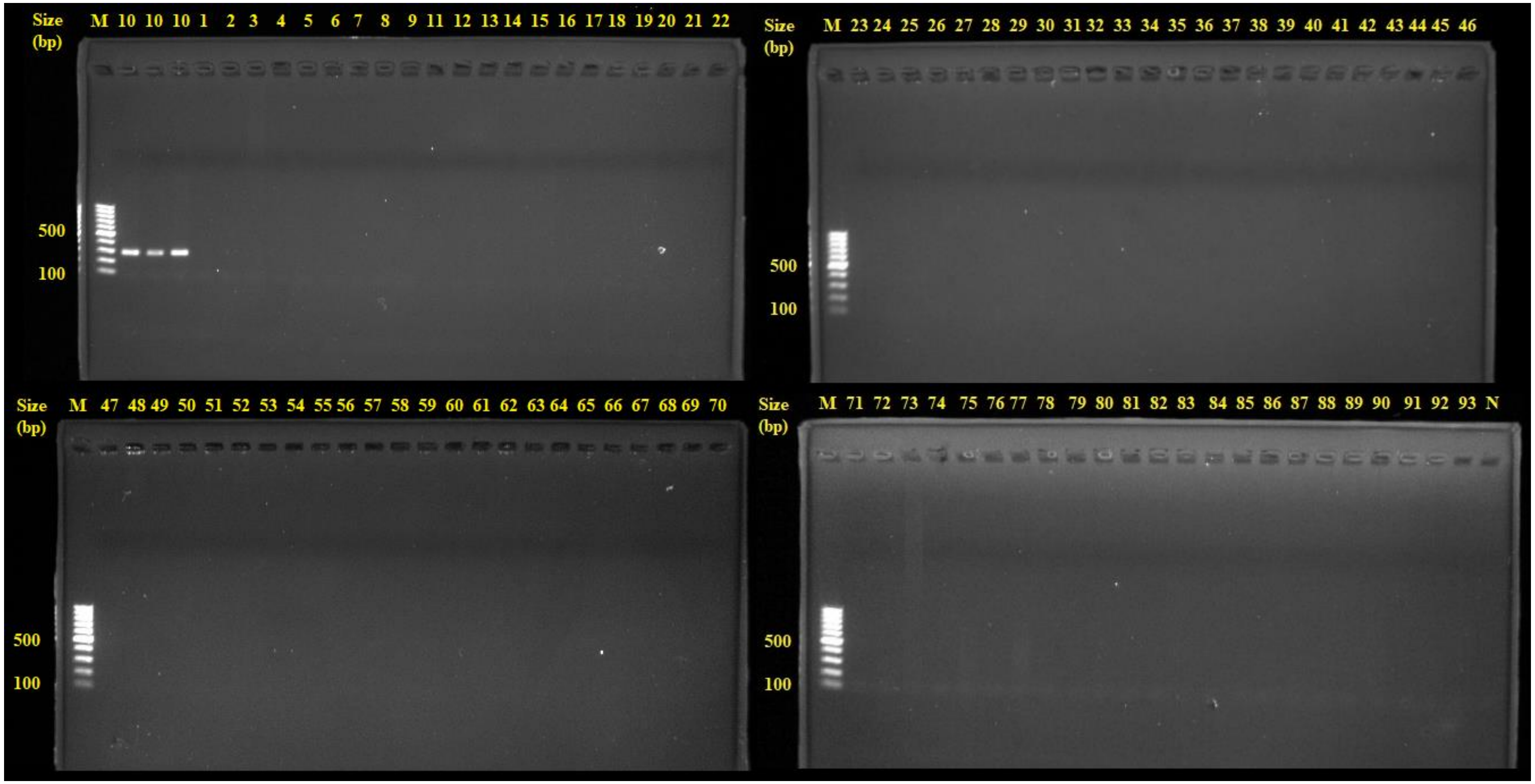

1 ***S. mokarran***
2 ***S. lewini***
3 ***S. zygaena***
4 ***C. longimanus***
5 ***L. nasus***
6 ***C. carcharias***
7 ***R. typus***
8 ***C. maximus***
9 ***C. falciformis***
10 ***A. pelagicus***
11 ***A. superciliosus***
12 ***A. vulpinus***
13 ***A. macrorhynchus***
14 ***A. nakayai***
15 ***A. platyrhynchus***
16 ***A. marmoratus***
17 ***C. albimarginatus***
18 ***C. altimus***
19 ***C. brachyurus***
20 ***C. brevipinna***
21 ***C. leucas***
22 ***C. limbatus***
23 ***C. melanopterus***
24 ***C. obscurus***
25 ***C. perezi***
26 ***C. plumbeus***
27 ***C. sorrah***
28 ***C. acus***
29 ***C. fabricii***
30 ***C. umbratile***
31 ***D. quadrispinosa***
32 ***E. cookei***
33 ***E. brachyurus***
34 ***E. decacuspidatus***
35 ***E. fusus***
36 ***E. molleri***
37 ***E. pusillus***
38 ***G. cuvier***
39 ***G. sauteri***
40 ***G. cirratum***
41 ***H. japanica***
42 ***H. perlo***
43 ***I. oxyrinchus***
44 ***I. paucus***
45 ***L. ditropis***
46 ***M. pelagios***
47 ***M. owstoni***
48 ***M. griseus***
49 ***M. manazo***
50 ***N. acutiden***
51 ***N. brevirostris***
52 ***O. maculatus***
53 ***P. xaniurus***
54 ***P. glauca***
55 ***P. habereri***
56 ***S. pacificus***
57 ***S. tudes***
58 ***S. brevirostris***
59 ***S. japonicus***
60 ***S. legnota***
61 ***T. kabeyai***
62 ***C. callorynchus***
63 ***C. acronotus***
64 ***C. amblyrhynchoides***
65 ***C. amblyrhynchos***
66 ***C. amboinensis***
67 ***C. galapagensis***
68 ***C. isodon***
69 ***C. macloti***
70 ***C. porosus***
71 ***Carcharias taurus***
72 ***Dalatias licha***
73 ***D. profundorum***
74 ***E. blochii***
75 ***G. galeus***
76 ***H. australiensis***
77 ***H. elongata***
78 ***H. griseus***
79 ***H. novaezealandiae***
80 ***L. temminckii***
81 ***L. macrorhinus***
82 ***M. californicus***
83 ***M. canis***
84 ***M. henlei***
85 ***M. lunulatus***
86 ***M. mosis***
87 ***M. mustelus***
88 ***M. punctulatus***
89 ***R. acutus***
90 ***R. longurio***
91 ***R. taylori***
92 ***S. laticaudus***
93 ***S. tiburo***
N **Negative Control**
M **100 bp DNA marker**
E **Empty**

Figure S4 Amplification results of all 93 species with species-specific PCR primers targeting bigeye thresher shark *Alopias superciliosus*.

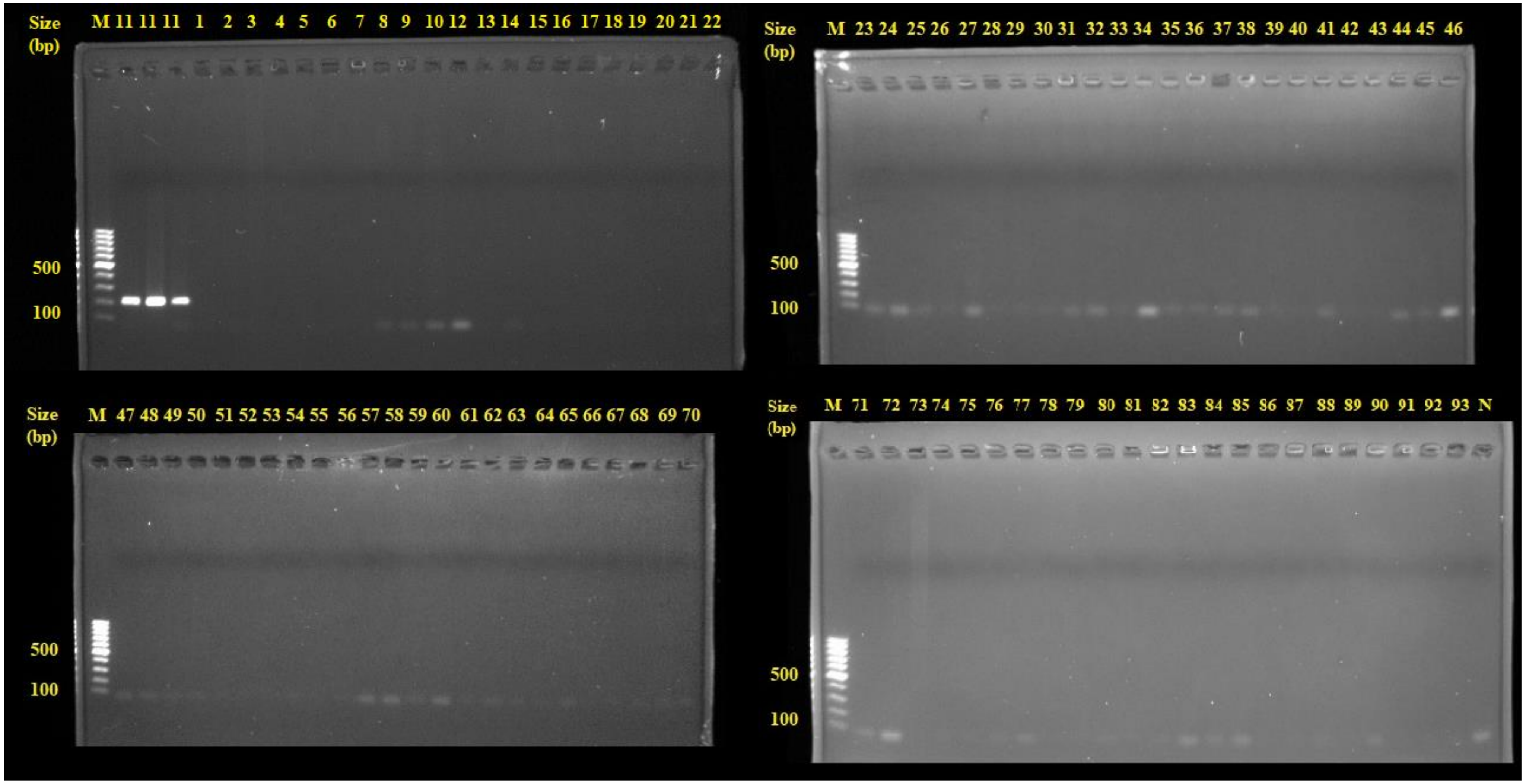

1 *S. mokarran*
2 *S. lewini*
3 *S. zygaena*
4 *C. longimanus*
5 *L. nasus*
6 *C. carcharias*
7 *R. typus*
8 *C. maximus*
9 *C. falciformis*
10 *A. pelagicus*
11 *A. superciliosus*
12 *A. vulpinus*
13 *A. macrorhynchus*
14 *A. nakayai*
15 *A. platyrhynchus*
16 *A. marmoratus*
17 *C. albimarginatus*
18 *C. altimus*
19 *C. brachyurus*
20 *C. brevipinna*
21 *C. leucas*
22 *C. limbatus*
23 *C. melanopterus*
24 *C. obscurus*
25 *C. perezi*
26 *C. plumbeus*
27 *C. sorrah*
28 *C. acus*
29 *C. fabricii*
30 *C. umbratile*
31 *D. quadrispinosa*
32 *E. cookei*
33 *E. brachyurus*
34 *E. decacuspidatus*
35 *E. fusus*
36 *E. molleri*
37 *E. pusillus*
38 *G. cuvier*
39 *G. sauteri*
40 *G. cirratum*
41 *H. japanica*
42 *H. perlo*
43 *I. oxyrinchus*
44 *I. paucus*
45 *L. ditropis*
46 *M. pelagios*
47 *M. owstoni*
48 *M. griseus*
49 *M. manazo*
50 *N. acutiden*
51 *N. brevirostris*
52 *O. maculatus*
53 *P. xaniurus*
54 *P. glauca*
55 *P. habereri*
56 *S. pacificus*
57 *S. tudes*
58 *S. brevirostris*
59 *S. japonicus*
60 *S. legnota*
61 *T. kabeyai*
62 *C. callorynchus*
63 *C. acronotus*
64 *C. amblyrhynchoides*
65 *C. amblyrhynchos*
66 *C. amboinensis*
67 *C. galapagensis*
68 *C. isodon*
69 *C. macloti*
70 *C. porosus*
71 *Carcharias taurus*
72 *Dalatias licha*
73 *D. profundorum*
74 *E. blochii*
75 *G. galeus*
76 *H. australiensis*
77 *H. elongata*
78 *H. griseus*
79 *H. novaezealandiae*
80 *L. temminckii*
81 *L. macrorhinus*
82 *M. californicus*
83 *M. canis*
84 *M. henlei*
85 *M. lunulatus*
86 *M. mosis*
87 *M. mustelus*
88 *M. punctulatus*
89 *R. acutus*
90 *R. longurio*
91 *R. taylori*
92 *S. laticaudus*
93 *S. tiburo*
N Negative Control
M 100 bp DNA marker
E Empty

Figure S5 Amplification results of all 93 species with species-specific PCR primers targeting common thresher shark *Alopias vulpinus*.

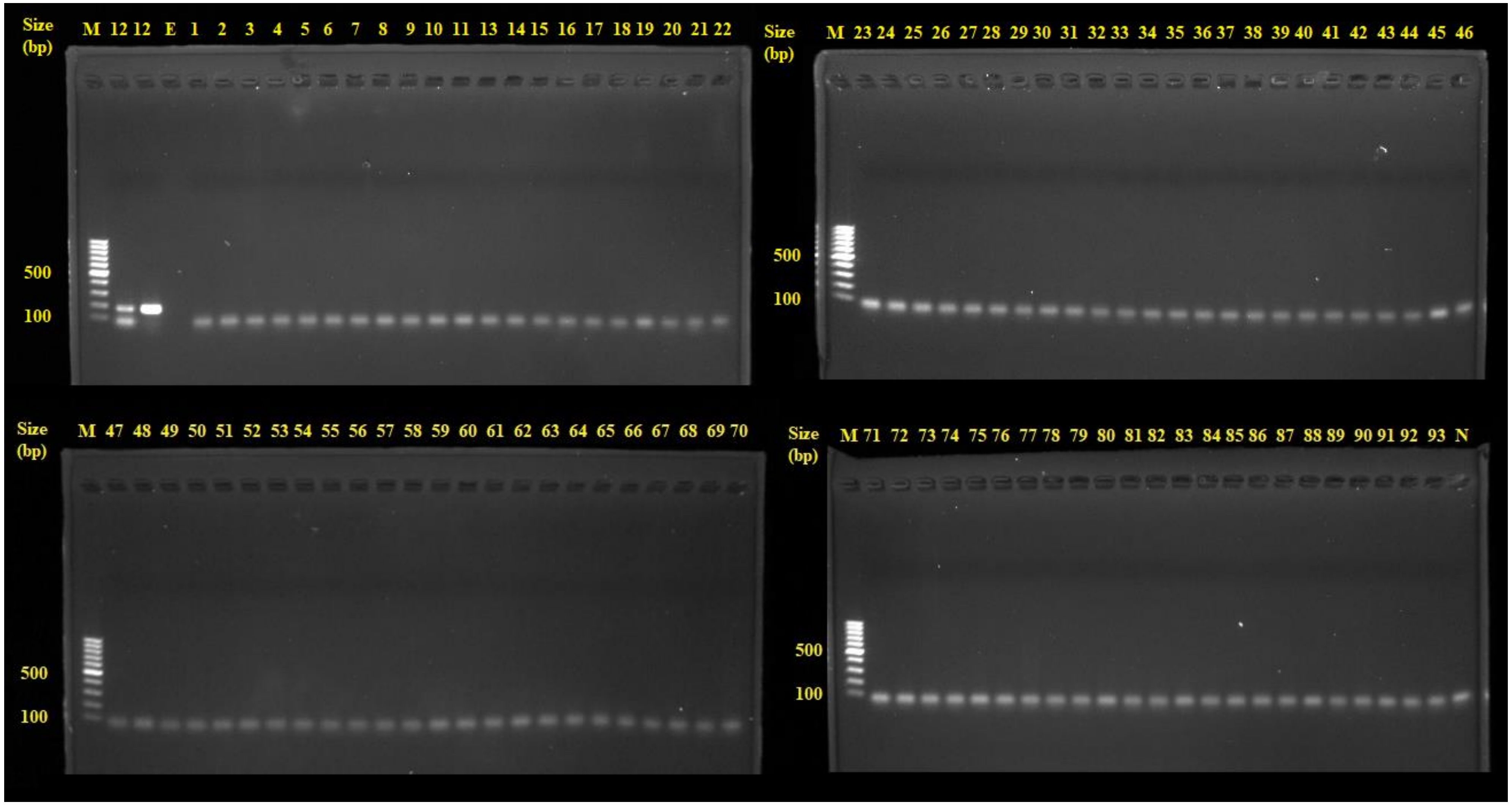

1 *S. mokarran*
2 *S. lewini*
3 *S. zygaena*
4 *C. longimanus*
5 *L. nasus*
6 *C. carcharias*
7 *R. typus*
8 *C. maximus*
9 *C. falciformis*
10 *A. pelagicus*
11 *A. superciliosus*
12 *A. vulpinus*
13 *A. macrorhynchus*
14 *A. nakayai*
15 *A. platyrhynchus*
16 *A. marmoratus*
17 *C. albimarginatus*
18 *C. altimus*
19 *C. brachyurus*
20 *C. brevipinna*
21 *C. leucas*
22 *C. limbatus*
23 *C. melanopterus*
24 *C. obscurus*
25 *C. perezi*
26 *C. plumbeus*
27 *C. sorrah*
28 *C. acus*
29 *C. fabricii*
30 *C. umbratile*
31 *D. quadrispinosa*
32 *E. cookei*
33 *E. brachyurus*
34 *E. decacuspidatus*
35 *E. fusus*
36 *E. molleri*
37 *E. pusillus*
38 *G. cuvier*
39 *G. sauteri*
40 *G. cirratum*
41 *H. japanica*
42 *H. perlo*
43 *I. oxyrinchus*
44 *I. paucus*
45 *L. ditropis*
46 *M. pelagios*
47 *M. owstoni*
48 *M. griseus*
49 *M. manazo*
50 *N. acutiden*
51 *N. brevirostris*
52 *O. maculatus*
53 *P. xaniurus*
54 *P. glauca*
55 *P. habereri*
56 *S. pacificus*
57 *S. tudes*
58 *S. brevirostris*
59 *S. japonicus*
60 *S. legnota*
61 *T. kabeyai*
62 *C. callorynchus*
63 *C. acronotus*
64 *C. amblyrhynchoides*
65 *C. amblyrhynchos*
66 *C. amboinensis*
67 *C. galapagensis*
68 *C. isodon*
69 *C. macloti*
70 *C. porosus*
71 *Carcharias taurus*
72 *Dalatias licha*
73 *D. profundorum*
74 *E. blochii*
75 *G. galeus*
76 *H. australiensis*
77 *H. elongata*
78 *H. griseus*
79 *H. novaezealandiae*
80 *L. temminckii*
81 *L. macrorhinus*
82 *M. californicus*
83 *M. canis*
84 *M. henlei*
85 *M. lunulatus*
86 *M. mosis*
87 *M. mustelus*
88 *M. punctulatus*
89 *R. acutus*
90 *R. longurio*
91 *R. taylori*
92 *S. laticaudus*
93 *S. tiburo*
N Negative Control
M 100 bp DNA marker
E Empty

Figure S6 Amplification results of all 93 species with species-specific PCR primers targeting great white shark *Carcharodon carcharias.*

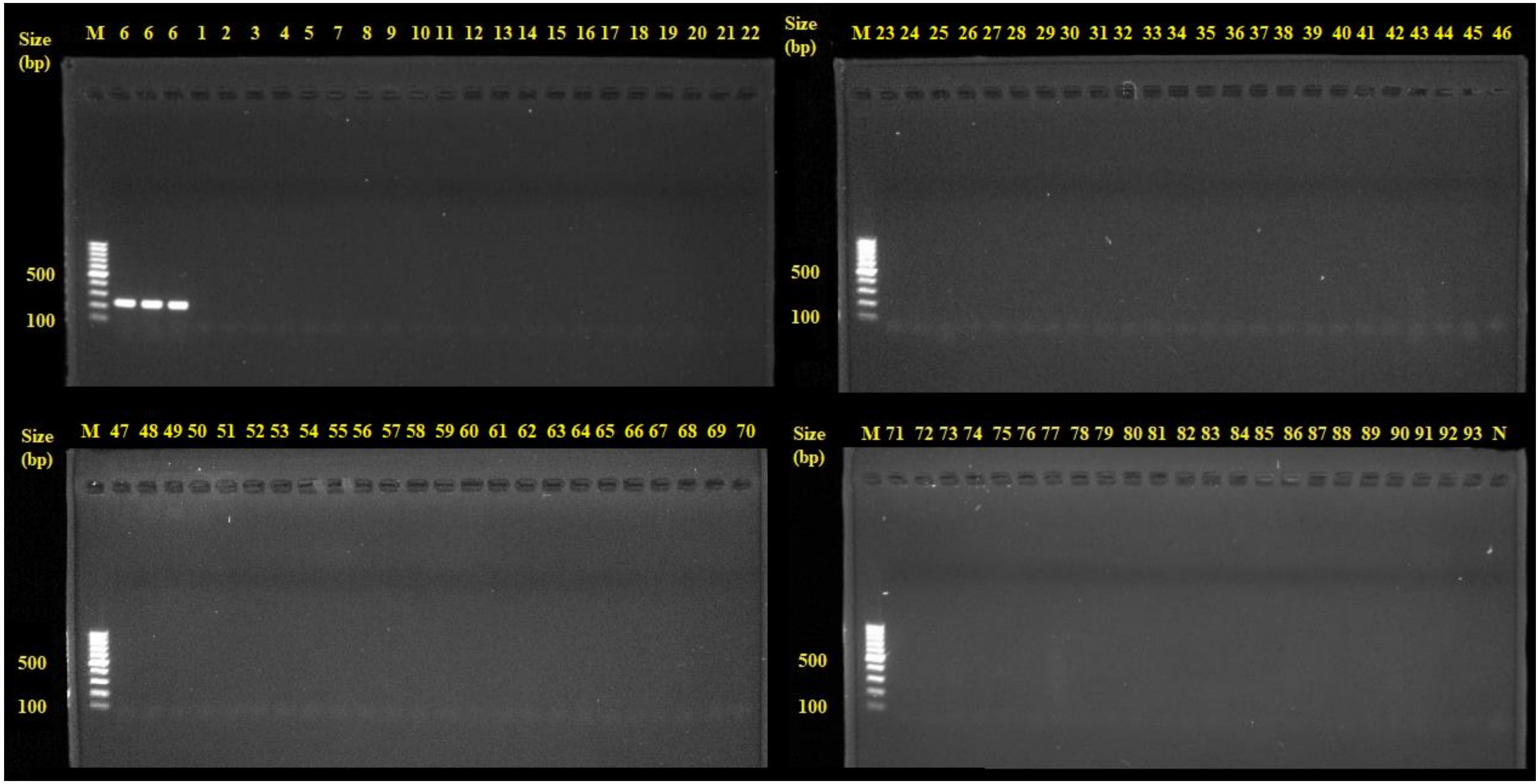

1 *S. mokarran*
2 *S. lewini*
3 *S. zygaena*
4 *C. longimanus*
5 *L. nasus*
6 *C. carcharias*
7 *R. typus*
8 *C. maximus*
9 *C. falciformis*
10 *A. pelagicus*
11 *A. superciliosus*
12 *A. vulpinus*
13 *A. macrorhynchus*
14 *A. nakayai*
15 *A. platyrhynchus*
16 *A. marmoratus*
17 *C. albimarginatus*
18 *C. altimus*
19 *C. brachyurus*
20 *C. brevipinna*
21 *C. leucas*
22 *C. limbatus*
23 *C. melanopterus*
24 *C. obscurus*
25 *C. perezi*
26 *C. plumbeus*
27 *C. sorrah*
28 *C. acus*
29 *C. fabricii*
30 *C. umbratile*
31 *D. quadrispinosa*
32 *E. cookei*
33 *E. brachyurus*
34 *E. decacuspidatus*
35 *E. fusus*
36 *E. molleri*
37 *E. pusillus*
38 *G. cuvier*
39 *G. sauteri*
40 *G. cirratum*
41 *H. japanica*
42 *H. perlo*
43 *I. oxyrinchus*
44 *I. paucus*
45 *L. ditropis*
46 *M. pelagios*
47 *M. owstoni*
48 *M. griseus*
49 *M. manazo*
50 *N. acutiden*
51 *N. brevirostris*
52 *O. maculatus*
53 *P. xaniurus*
54 *P. glauca*
55 *P. habereri*
56 *S. pacificus*
57 *S. tudes*
58 *S. brevirostris*
59 *S. japonicus*
60 *S. legnota*
61 *T. kabeyai*
62 *C. callorynchus*
63 *C. acronotus*
64 *C. amblyrhynchoides*
65 *C. amblyrhynchos*
66 *C. amboinensis*
67 *C. galapagensis*
68 *C. isodon*
69 *C. macloti*
70 *C. porosus*
71 *Carcharias taurus*
72 *Dalatias licha*
73 *D. profundorum*
74 *E. blochii*
75 *G. galeus*
76 *H. australiensis*
77 *H. elongata*
78 *H. griseus*
79 *H. novaezealandiae*
80 *L. temminckii*
81 *L. macrorhinus*
82 *M. californicus*
83 *M. canis*
84 *M. henlei*
85 *M. lunulatus*
86 *M. mosis*
87 *M. mustelus*
88 *M. punctulatus*
89 *R. acutus*
90 *R. longurio*
91 *R. taylori*
92 *S. laticaudus*
93 *S. tiburo*
N Negative Control
M 100 bp DNA marker
E Empty

Figure S7 Amplification results of all 93 species with species-specific PCR primers targeting silky shark *Carcharhinus falciformis*.

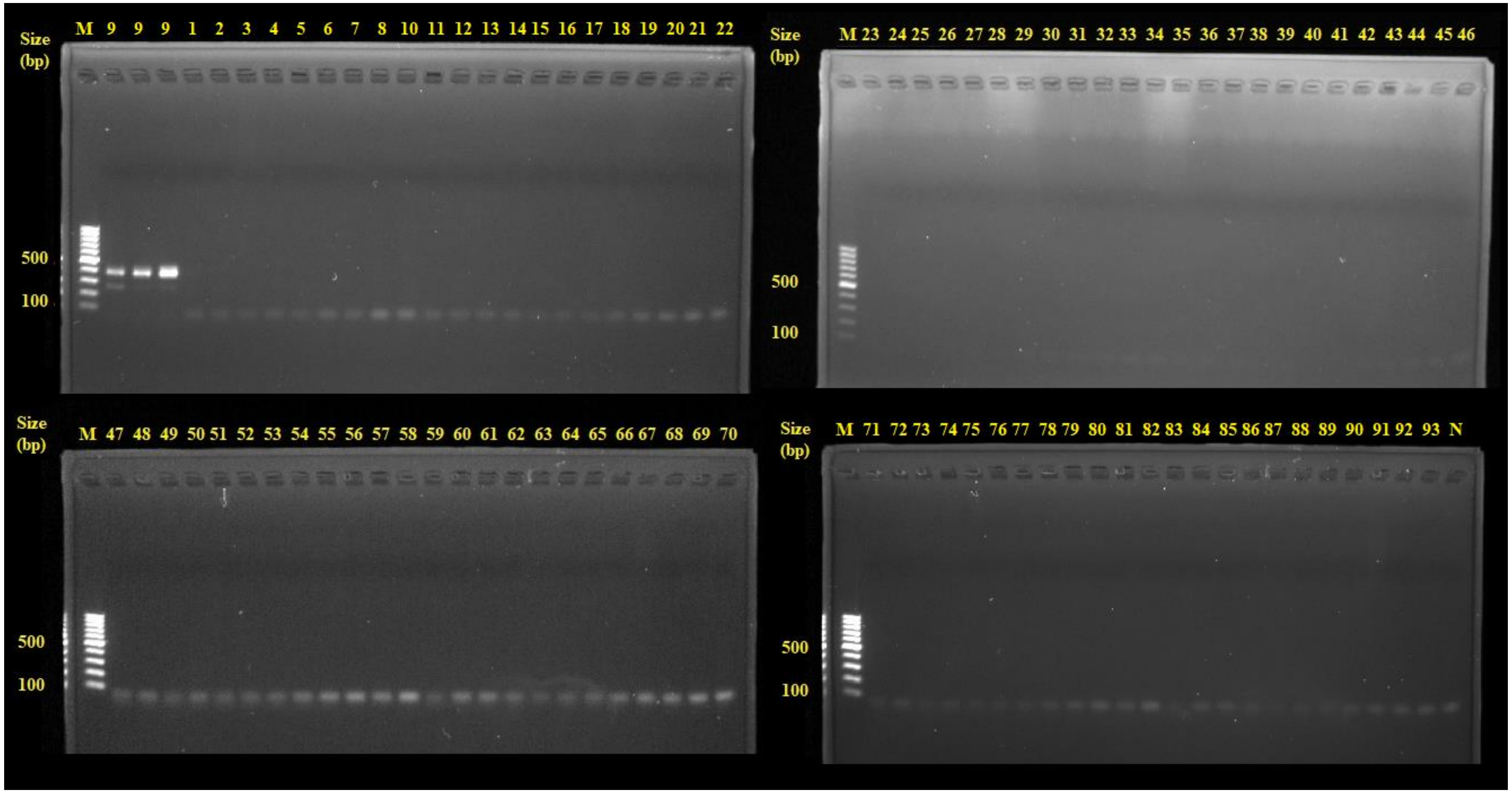

1 ***S. mokarran***
2 ***S. lewini***
3 ***S. zygaena***
4 ***C. longimanus***
5 ***L. nasus***
6 ***C. carcharias***
7 ***R. typus***
8 ***C. maximus***
9 ***C. falciformis***
10 ***A. pelagicus***
11 ***A. superciliosus***
12 ***A. vulpinus***
13 ***A. macrorhynchus***
14 ***A. nakayai***
15 ***A. platyrhynchus***
16 ***A. marmoratus***
17 ***C. albimarginatus***
18 ***C. altimus***
19 ***C. brachyurus***
20 ***C. brevipinna***
21 ***C. leucas***
22 ***C. limbatus***
23 ***C. melanopterus***
24 ***C. obscurus***
25 ***C. perezi***
26 ***C. plumbeus***
27 ***C. sorrah***
28 ***C. acus***
29 ***C. fabricii***
30 ***C. umbratile***
31 ***D. quadrispinosa***
32 ***E. cookei***
33 ***E. brachyurus***
34 ***E. decacuspidatus***
35 ***E. fusus***
36 ***E. molleri***
37 ***E. pusillus***
38 ***G. cuvier***
39 ***G. sauteri***
40 ***G. cirratum***
41 ***H. japanica***
42 ***H. perlo***
43 ***I. oxyrinchus***
44 ***I. paucus***
45 ***L. ditropis***
46 ***M. pelagios***
47 ***M. owstoni***
48 ***M. griseus***
49 ***M. manazo***
50 ***N. acutiden***
51 ***N. brevirostris***
52 ***O. maculatus***
53 ***P. xaniurus***
54 ***P. glauca***
55 ***P. habereri***
56 ***S. pacificus***
57 ***S. tudes***
58 ***S. brevirostris***
59 ***S. japonicus***
60 ***S. legnota***
61 ***T. kabeyai***
62 ***C. callorynchus***
63 ***C. acronotus***
64 ***C. amblyrhynchoides***
65 ***C. amblyrhynchos***
66 ***C. amboinensis***
67 ***C. galapagensis***
68 ***C. isodon***
69 ***C. macloti***
70 ***C. porosus***
71 ***Carcharias taurus***
72 ***Dalatias licha***
73 ***D. profundorum***
74 ***E. blochii***
75 ***G. galeus***
76 ***H. australiensis***
77 ***H. elongata***
78 ***H. griseus***
79 ***H. novaezealandiae***
80 ***L. temminckii***
81 ***L. macrorhinus***
82 ***M. californicus***
83 ***M. canis***
84 ***M. henlei***
85 ***M. lunulatus***
86 ***M. mosis***
87 ***M. mustelus***
88 ***M. punctulatus***
89 ***R. acutus***
90 ***R. longurio***
91 ***R. taylori***
92 ***S. laticaudus***
93 ***S. tiburo***
N **Negative Control**
M **100 bp DNA marker**
E **Empty**

Figure S8 Amplification results of all 93 species with species-specific PCR primers targeting oceanic whitetip shark *Carcharhinus longimanus*.

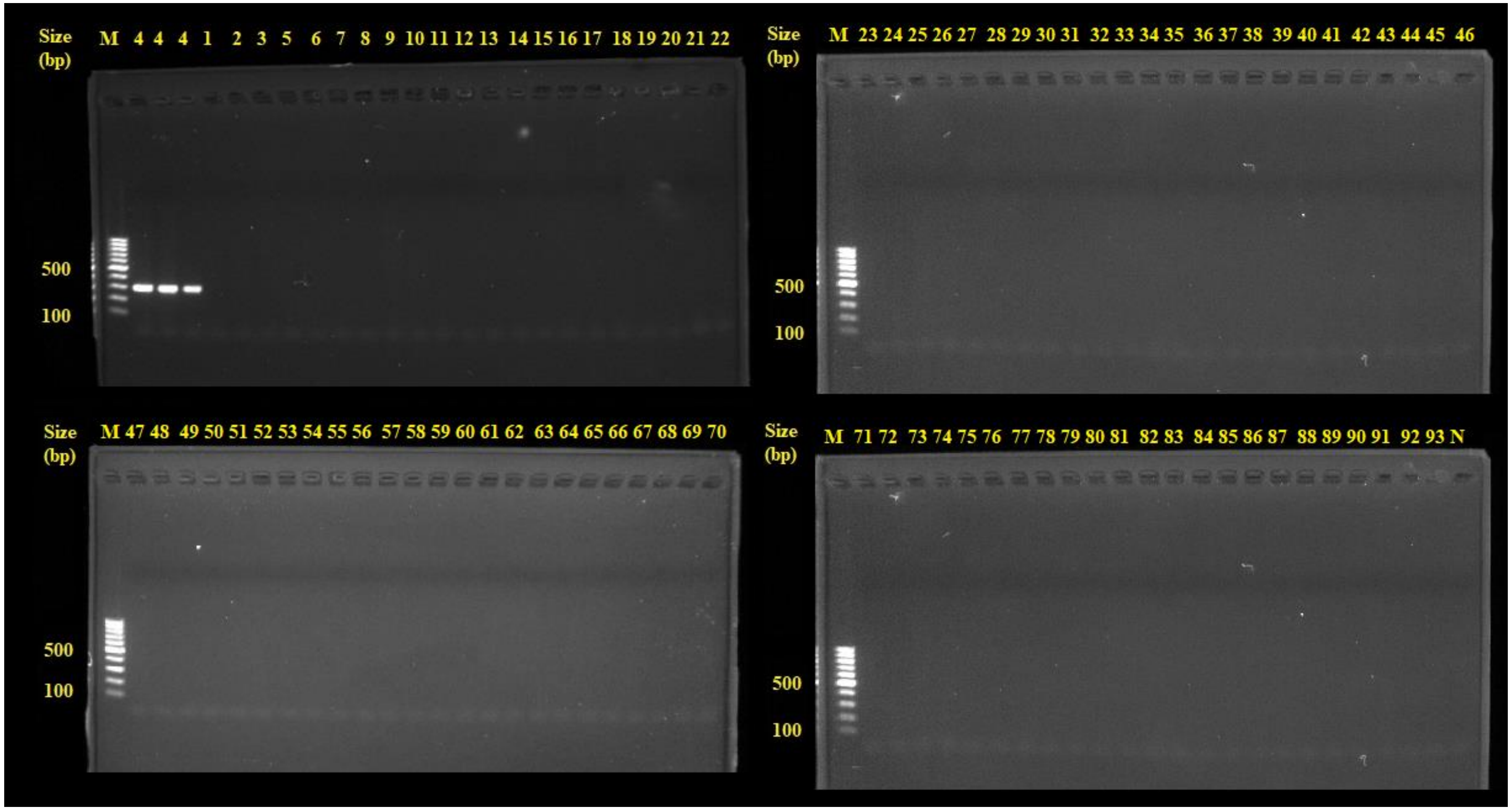

1 ***S. mokarran***
2 ***S. lewini***
3 ***S. zygaena***
4 ***C. longimanus***
5 ***L. nasus***
6 ***C. carcharias***
7 ***R. typus***
8 ***C. maximus***
9 ***C. falciformis***
10 ***A. pelagicus***
11 ***A. superciliosus***
12 ***A. vulpinus***
13 ***A. macrorhynchus***
14 ***A. nakayai***
15 ***A. platyrhynchus***
16 ***A. marmoratus***
17 ***C. albimarginatus***
18 ***C. altimus***
19 ***C. brachyurus***
20 ***C. brevipinna***
21 ***C. leucas***
22 ***C. limbatus***
23 ***C. melanopterus***
24 ***C. obscurus***
25 ***C. perezi***
26 ***C. plumbeus***
27 ***C. sorrah***
28 ***C. acus***
29 ***C. fabricii***
30 ***C. umbratile***
31 ***D. quadrispinosa***
32 ***E. cookei***
33 ***E. brachyurus***
34 ***E. decacuspidatus***
35 ***E. fusus***
36 ***E. molleri***
37 ***E. pusillus***
38 ***G. cuvier***
39 ***G. sauteri***
40 ***G. cirratum***
41 ***H. japanica***
42 ***H. perlo***
43 ***I. oxyrinchus***
44 ***I. paucus***
45 ***L. ditropis***
46 ***M. pelagios***
47 ***M. owstoni***
48 ***M. griseus***
49 ***M. manazo***
50 ***N. acutiden***
51 ***N. brevirostris***
52 ***O. maculatus***
53 ***P. xaniurus***
54 ***P. glauca***
55 ***P. habereri***
56 ***S. pacificus***
57 ***S. tudes***
58 ***S. brevirostris***
59 ***S. japonicus***
60 ***S. legnota***
61 ***T. kabeyai***
62 ***C. callorynchus***
63 ***C. acronotus***
64 ***C. amblyrhynchoides***
65 ***C. amblyrhynchos***
66 ***C. amboinensis***
67 ***C. galapagensis***
68 ***C. isodon***
69 ***C. macloti***
70 ***C. porosus***
71 ***Carcharias taurus***
72 ***Dalatias licha***
73 ***D. profundorum***
74 ***E. blochii***
75 ***G. galeus***
76 ***H. australiensis***
77 ***H. elongata***
78 ***H. griseus***
79 ***H. novaezealandiae***
80 ***L. temminckii***
81 ***L. macrorhinus***
82 ***M. californicus***
83 ***M. canis***
84 ***M. henlei***
85 ***M. lunulatus***
86 ***M. mosis***
87 ***M. mustelus***
88 ***M. punctulatus***
89 ***R. acutus***
90 ***R. longurio***
91 ***R. taylori***
92 ***S. laticaudus***
93 ***S. tiburo***
N **Negative Control**
M **100 bp DNA marker**
E **Empty**

Figure S9 Amplification results of all 93 species with species-specific PCR primers targeting basking shark *Cetorhinus maximus*.

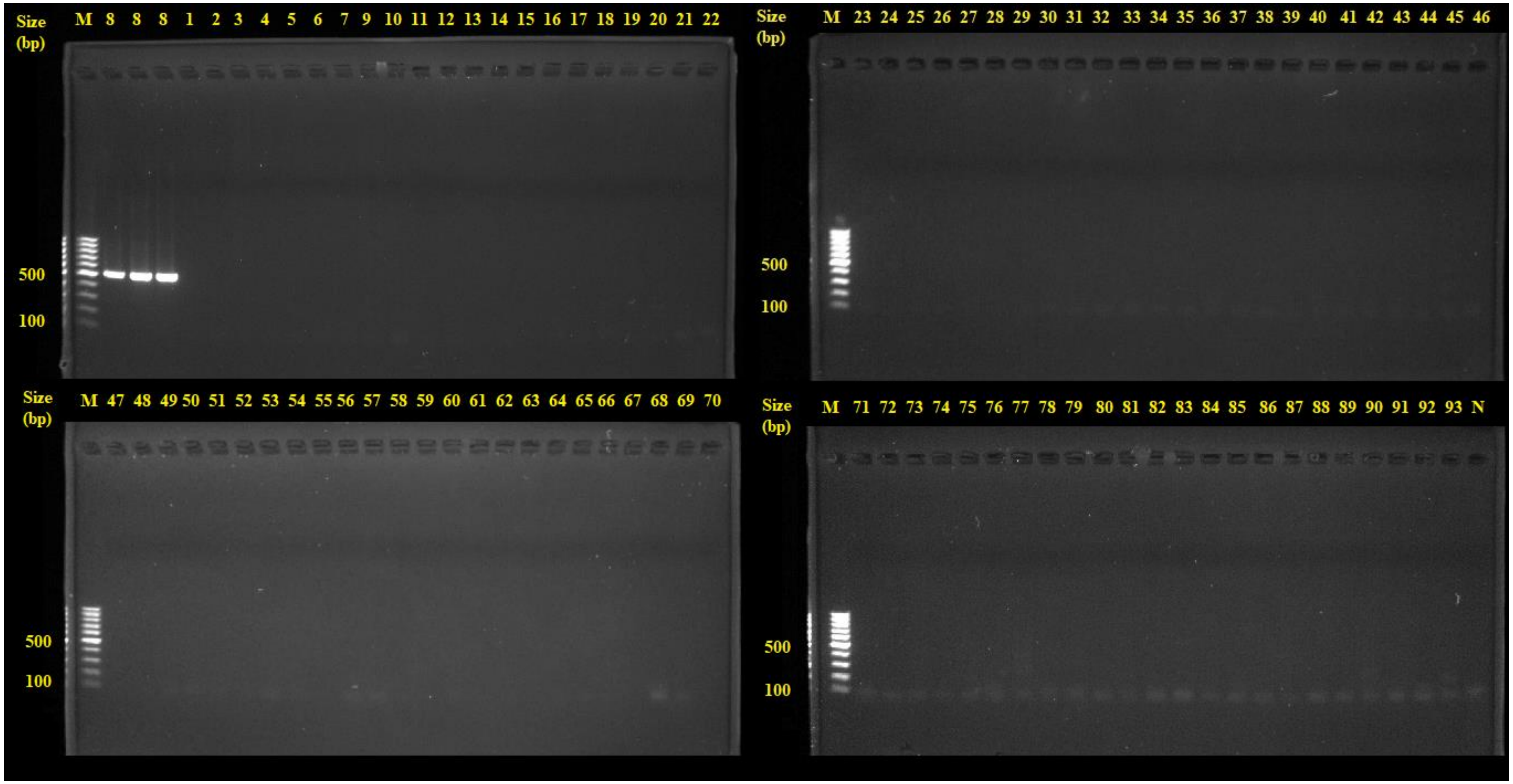

1 ***S. mokarran***
2 ***S. lewini***
3 ***S. zygaena***
4 ***C. longimanus***
5 ***L. nasus***
6 ***C. carcharias***
7 ***R. typus***
8 ***C. maximus***
9 ***C. falciformis***
10 ***A. pelagicus***
11 ***A. superciliosus***
12 ***A. vulpinus***
13 ***A. macrorhynchus***
14 ***A. nakayai***
15 ***A. platyrhynchus***
16 ***A. marmoratus***
17 ***C. albimarginatus***
18 ***C. altimus***
19 ***C. brachyurus***
20 ***C. brevipinna***
21 ***C. leucas***
22 ***C. limbatus***
23 ***C. melanopterus***
24 ***C. obscurus***
25 ***C. perezi***
26 ***C. plumbeus***
27 ***C. sorrah***
28 ***C. acus***
29 ***C. fabricii***
30 ***C. umbratile***
31 ***D. quadrispinosa***
32 ***E. cookei***
33 ***E. brachyurus***
34 ***E. decacuspidatus***
35 ***E. fusus***
36 ***E. molleri***
37 ***E. pusillus***
38 ***G. cuvier***
39 ***G. sauteri***
40 ***G. cirratum***
41 ***H. japanica***
42 ***H. perlo***
43 ***I. oxyrinchus***
44 ***I. paucus***
45 ***L. ditropis***
46 ***M. pelagios***
47 ***M. owstoni***
48 ***M. griseus***
49 ***M. manazo***
50 ***N. acutiden***
51 ***N. brevirostris***
52 ***O. maculatus***
53 ***P. xaniurus***
54 ***P. glauca***
55 ***P. habereri***
56 ***S. pacificus***
57 ***S. tudes***
58 ***S. brevirostris***
59 ***S. japonicus***
60 ***S. legnota***
61 ***T. kabeyai***
62 ***C. callorynchus***
63 ***C. acronotus***
64 ***C. amblyrhynchoides***
65 ***C. amblyrhynchos***
66 ***C. amboinensis***
67 ***C. galapagensis***
68 ***C. isodon***
69 ***C. macloti***
70 ***C. porosus***
71 ***Carcharias taurus***
72 ***Dalatias licha***
73 ***D. profundorum***
74 ***E. blochii***
75 ***G. galeus***
76 ***H. australiensis***
77 ***H. elongata***
78 ***H. griseus***
79 ***H. novaezealandiae***
80 ***L. temminckii***
81 ***L. macrorhinus***
82 ***M. californicus***
83 ***M. canis***
84 ***M. henlei***
85 ***M. lunulatus***
86 ***M. mosis***
87 ***M. mustelus***
88 ***M. punctulatus***
89 ***R. acutus***
90 ***R. longurio***
91 ***R. taylori***
92 ***S. laticaudus***
93 ***S. tiburo***
N **Negative Control**
M **100 bp DNA marker**
E **Empty**

Figure S10 Amplification results of all 93 species with species-specific PCR primers targeting porbeagle shark *Lamna nasus*.

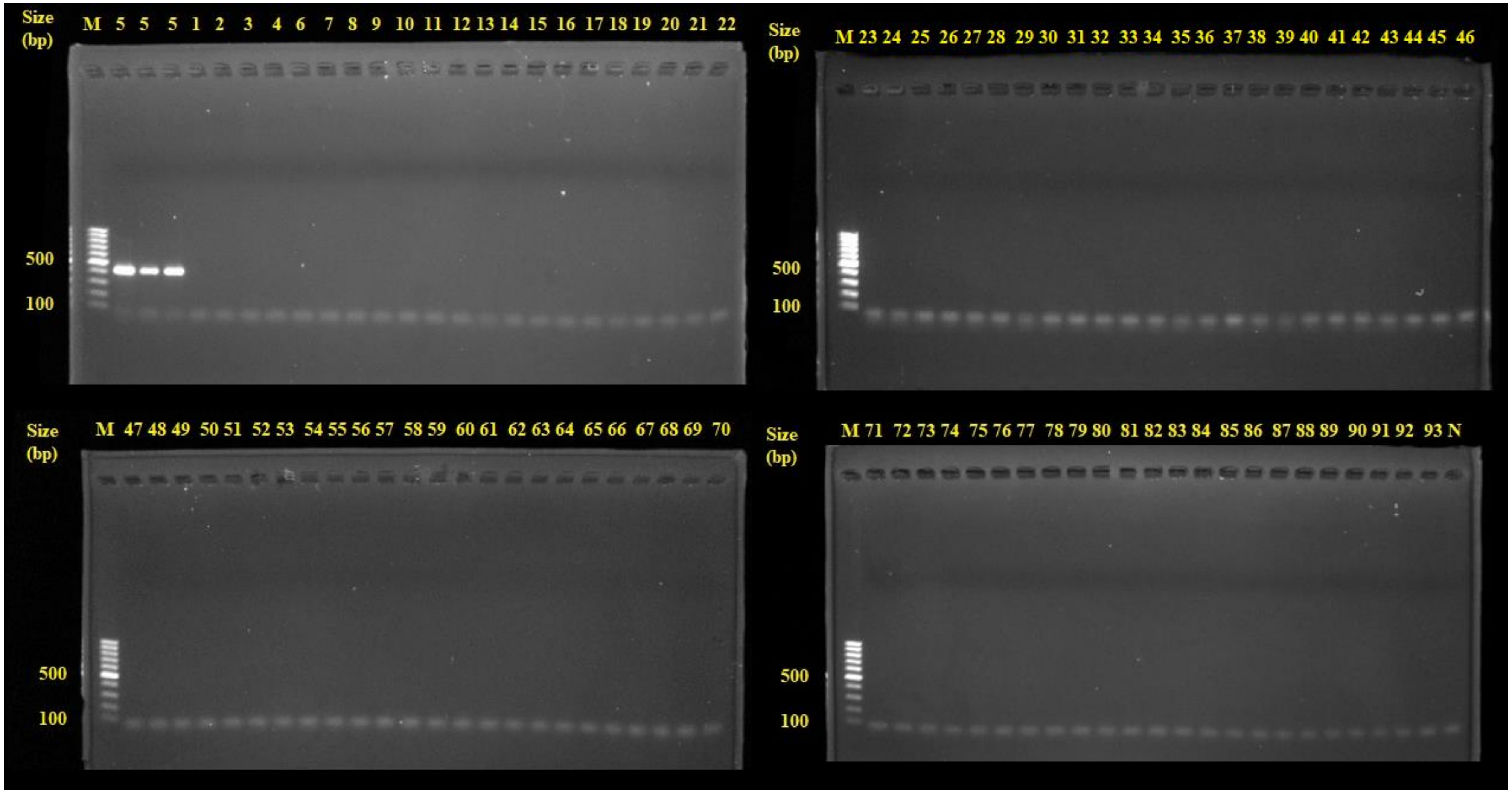

1 ***S. mokarran***
2 ***S. lewini***
3 ***S. zygaena***
4 ***C. longimanus***
5 ***L. nasus***
6 ***C. carcharias***
7 ***R. typus***
8 ***C. maximus***
9 ***C. falciformis***
10 ***A. pelagicus***
11 ***A. superciliosus***
12 ***A. vulpinus***
13 ***A. macrorhynchus***
14 ***A. nakayai***
15 ***A. platyrhynchus***
16 ***A. marmoratus***
17 ***C. albimarginatus***
18 ***C. altimus***
19 ***C. brachyurus***
20 ***C. brevipinna***
21 ***C. leucas***
22 ***C. limbatus***
23 ***C. melanopterus***
24 ***C. obscurus***
25 ***C. perezi***
26 ***C. plumbeus***
27 ***C. sorrah***
28 ***C. acus***
29 ***C. fabricii***
30 ***C. umbratile***
31 ***D. quadrispinosa***
32 ***E. cookei***
33 ***E. brachyurus***
34 ***E. decacuspidatus***
35 ***E. fusus***
36 ***E. molleri***
37 ***E. pusillus***
38 ***G. cuvier***
39 ***G. sauteri***
40 ***G. cirratum***
41 ***H. japanica***
42 ***H. perlo***
43 ***I. oxyrinchus***
44 ***I. paucus***
45 ***L. ditropis***
46 ***M. pelagios***
47 ***M. owstoni***
48 ***M. griseus***
49 ***M. manazo***
50 ***N. acutiden***
51 ***N. brevirostris***
52 ***O. maculatus***
53 ***P. xaniurus***
54 ***P. glauca***
55 ***P. habereri***
56 ***S. pacificus***
57 ***S. tudes***
58 ***S. brevirostris***
59 ***S. japonicus***
60 ***S. legnota***
61 ***T. kabeyai***
62 ***C. callorynchus***
63 ***C. acronotus***
64 ***C. amblyrhynchoides***
65 ***C. amblyrhynchos***
66 ***C. amboinensis***
67 ***C. galapagensis***
68 ***C. isodon***
69 ***C. macloti***
70 ***C. porosus***
71 ***Carcharias taurus***
72 ***Dalatias licha***
73 ***D. profundorum***
74 ***E. blochii***
75 ***G. galeus***
76 ***H. australiensis***
77 ***H. elongata***
78 ***H. griseus***
79 ***H. novaezealandiae***
80 ***L. temminckii***
81 ***L. macrorhinus***
82 ***M. californicus***
83 ***M. canis***
84 ***M. henlei***
85 ***M. lunulatus***
86 ***M. mosis***
87 ***M. mustelus***
88 ***M. punctulatus***
89 ***R. acutus***
90 ***R. longurio***
91 ***R. taylori***
92 ***S. laticaudus***
93 ***S. tiburo***
N Negative Control
M 100 bp DNA marker
E Empty

Figure S11 Amplification results of all 93 species with species-specific PCR primers targeting whale shark *Rhincodon typus*.

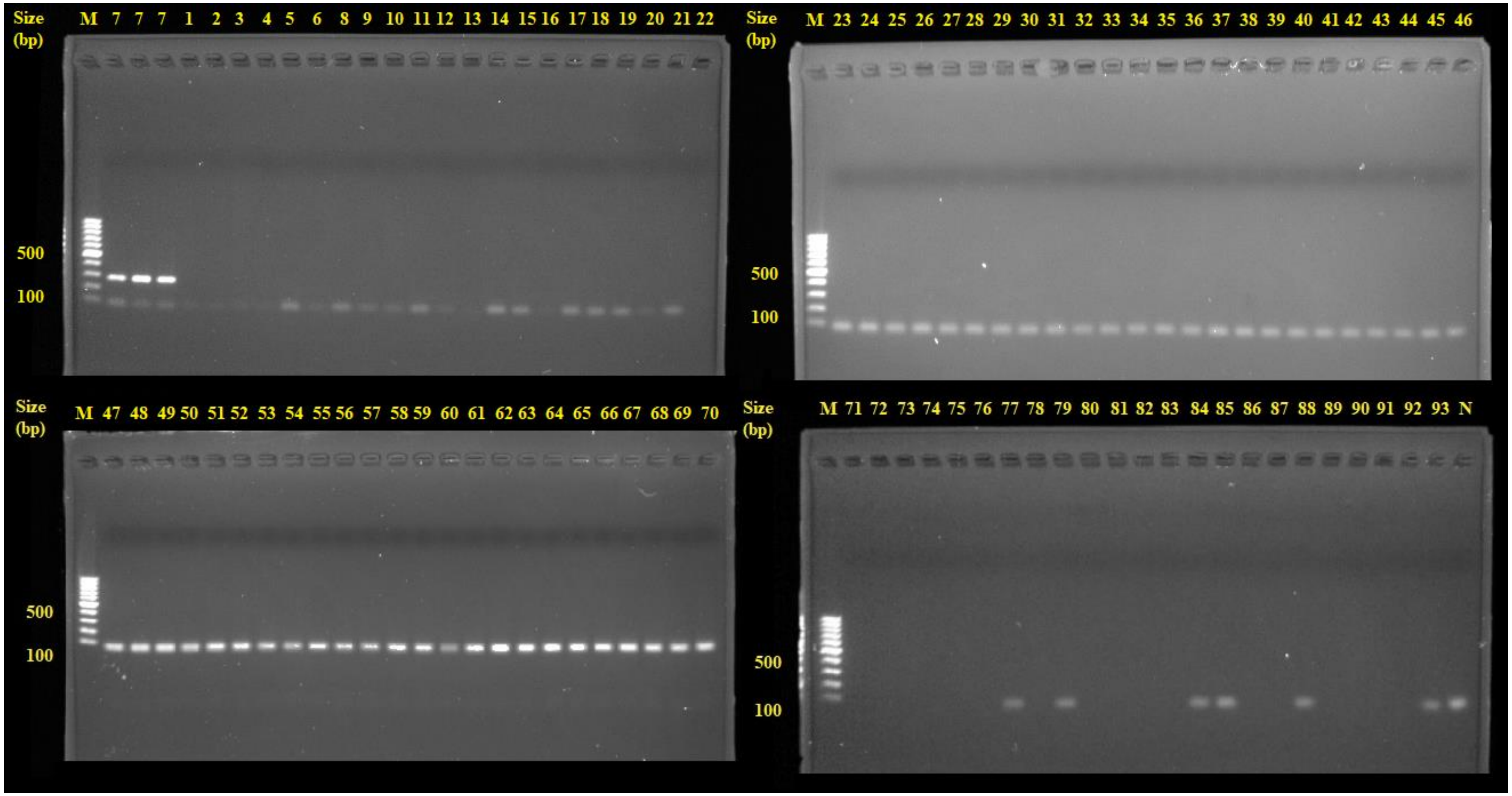

1 ***S. mokarran***
2 ***S. lewini***
3 ***S. zygaena***
4 ***C. longimanus***
5 ***L. nasus***
6 ***C. carcharias***
7 ***R. typus***
8 ***C. maximus***
9 ***C. falciformis***
10 ***A. pelagicus***
11 ***A. superciliosus***
12 ***A. vulpinus***
13 ***A. macrorhynchus***
14 ***A. nakayai***
15 ***A. platyrhynchus***
16 ***A. marmoratus***
17 ***C. albimarginatus***
18 ***C. altimus***
19 ***C. brachyurus***
20 ***C. brevipinna***
21 ***C. leucas***
22 ***C. limbatus***
23 ***C. melanopterus***
24 ***C. obscurus***
25 ***C. perezi***
26 ***C. plumbeus***
27 ***C. sorrah***
28 ***C. acus***
29 ***C. fabricii***
30 ***C. umbratile***
31 ***D. quadrispinosa***
32 ***E. cookei***
33 ***E. brachyurus***
34 ***E. decacuspidatus***
35 ***E. fusus***
36 ***E. molleri***
37 ***E. pusillus***
38 ***G. cuvier***
39 ***G. sauteri***
40 ***G. cirratum***
41 ***H. japanica***
42 ***H. perlo***
43 ***I. oxyrinchus***
44 ***I. paucus***
45 ***L. ditropis***
46 ***M. pelagios***
47 ***M. owstoni***
48 ***M. griseus***
49 ***M. manazo***
50 ***N. acutiden***
51 ***N. brevirostris***
52 ***O. maculatus***
53 ***P. xaniurus***
54 ***P. glauca***
55 ***P. habereri***
56 ***S. pacificus***
57 ***S. tudes***
58 ***S. brevirostris***
59 ***S. japonicus***
60 ***S. legnota***
61 ***T. kabeyai***
62 ***C. callorynchus***
63 ***C. acronotus***
64 ***C. amblyrhynchoides***
65 ***C. amblyrhynchos***
66 ***C. amboinensis***
67 ***C. galapagensis***
68 ***C. isodon***
69 ***C. macloti***
70 ***C. porosus***
71 ***Carcharias taurus***
72 ***Dalatias licha***
73 ***D. profundorum***
74 ***E. blochii***
75 ***G. galeus***
76 ***H. australiensis***
77 ***H. elongata***
78 ***H. griseus***
79 ***H. novaezealandiae***
80 ***L. temminckii***
81 ***L. macrorhinus***
82 ***M. californicus***
83 ***M. canis***
84 ***M. henlei***
85 ***M. lunulatus***
86 ***M. mosis***
87 ***M. mustelus***
88 ***M. punctulatus***
89 ***R. acutus***
90 ***R. longurio***
91 ***R. taylori***
92 ***S. laticaudus***
93 ***S. tiburo***
N **Negative Control**
M **100 bp DNA marker**
E **Empty**

Figure S12 Amplification results of all 93 species with species-specific PCR primers targeting scalloped hammerhead shark *Sphyrna lewini*.

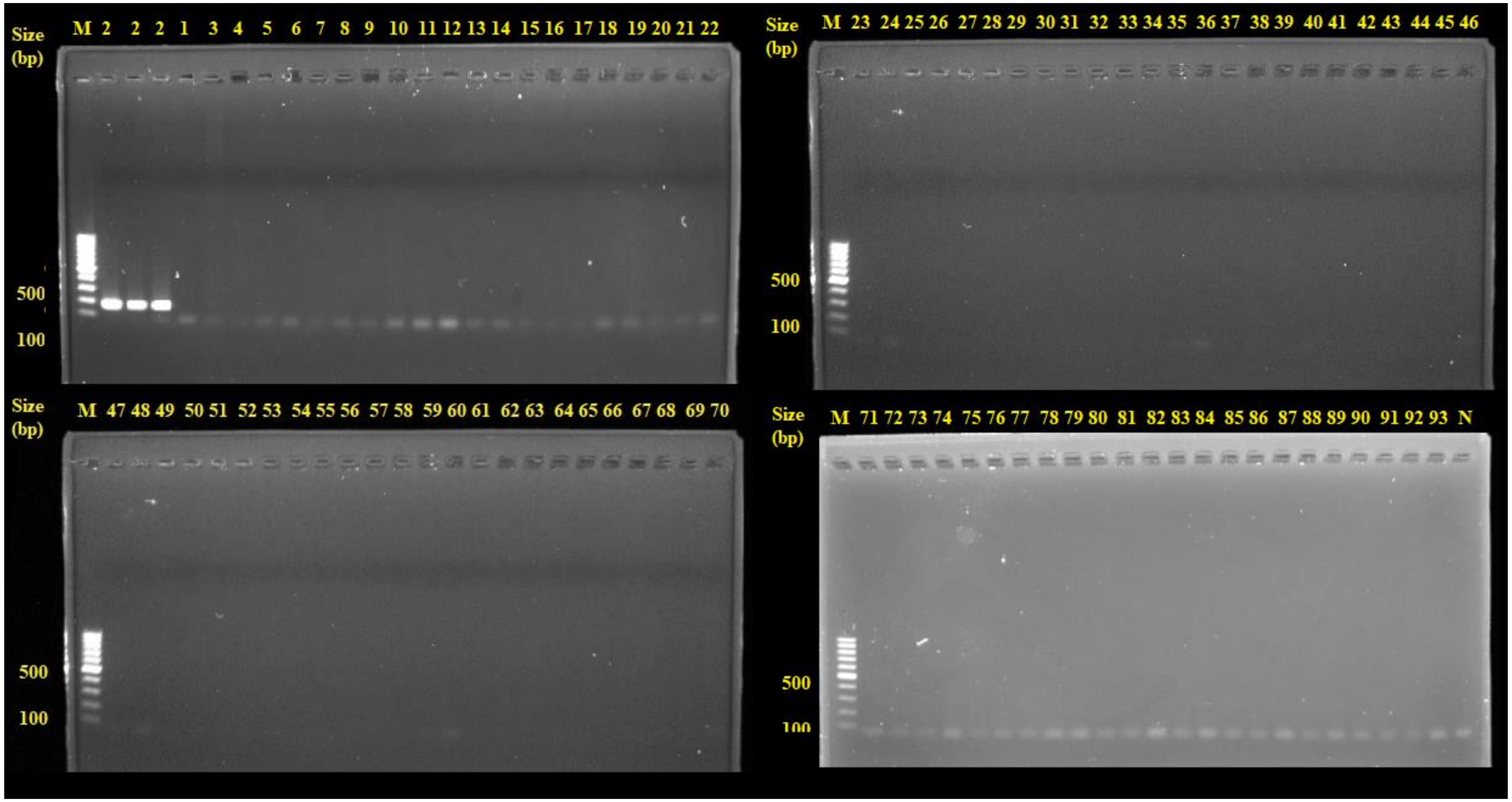

1 ***S. mokarran***	17 ***C. albimarginatus***	33 ***E. brachyurus***	49 ***M. manazo***	65 ***C. amblyrhynchos***	81 ***L. macrorhinus***
2 ***S. lewini***	18 ***C. altimus***	34 ***E. decacuspidatus***	50 ***N. acutiden***	66 ***C. amboinensis***	82 ***M. californicus***
3 ***S. zygaena***	19 ***C. brachyurus***	35 ***E. fusus***	51 ***N. brevirostris***	67 ***C. galapagensis***	83 ***M. canis***
4 ***C. longimanus***	20 ***C. brevipinna***	36 ***E. molleri***	52 ***O. maculatus***	68 ***C. isodon***	84 ***M. henlei***
5 ***L. nasus***	21 ***C. leucas***	37 ***E. pusillus***	53 ***P. xaniurus***	69 ***C. macloti***	85 ***M. lunulatus***
6 ***C. carcharias***	22 ***C. limbatus***	38 ***G. cuvier***	54 ***P. glauca***	70 ***C. porosus***	86 ***M. mosis***
7 ***R. typus***	23 ***C. melanopterus***	39 ***G. sauteri***	55 ***P. habereri***	71 ***Carcharias taurus***	87 ***M. mustelus***
8 ***C. maximus***	24 ***C. obscurus***	40 ***G. cirratum***	56 ***S. pacificus***	72 ***Dalatias licha***	88 ***M. punctulatus***
9 ***C. falciformis***	25 ***C. perezi***	41 ***H. japanica***	57 ***S. tudes***	73 ***D. profundorum***	89 ***R. acutus***
10 ***A. pelagicus***	26 ***C. plumbeus***	42 ***H. perlo***	58 ***S. brevirostris***	74 ***E. blochii***	90 ***R. longurio***
11 ***A. superciliosus***	27 ***C. sorrah***	43 ***I. oxyrinchus***	59 ***S. japonicus***	75 ***G. galeus***	91 ***R. taylori***
12 ***A. vulpinus***	28 ***C. acus***	44 ***I. paucus***	60 ***S. legnota***	76 ***H. australiensis***	92 ***S. laticaudus***
13 ***A. macrorhynchus***	29 ***C. fabricii***	45 ***L. ditropis***	61 ***T. kabeyai***	77 ***H. elongata***	93 ***S. tiburo***
14 ***A. nakayai***	30 ***C. umbratile***	46 ***M. pelagios***	62 ***C. callorynchus***	78 ***H. griseus***	**N Negative Control**
15 ***A. platyrhynchus***	31 ***D. quadrispinosa***	47 ***M. owstoni***	63 ***C. acronotus***	79 ***H. novaezealandiae***	**M 100 bp DNA marker**
16 ***A. marmoratus***	32 ***E. cookei***	48 ***M. griseus***	64 ***C. amblyrhynchoides***	80 ***L. temminckii***	**E Empty**

Figure S13 Amplification results of all 93 species with species-specific PCR primers targeting great hammerhead shark *Sphyrna mokarran.*

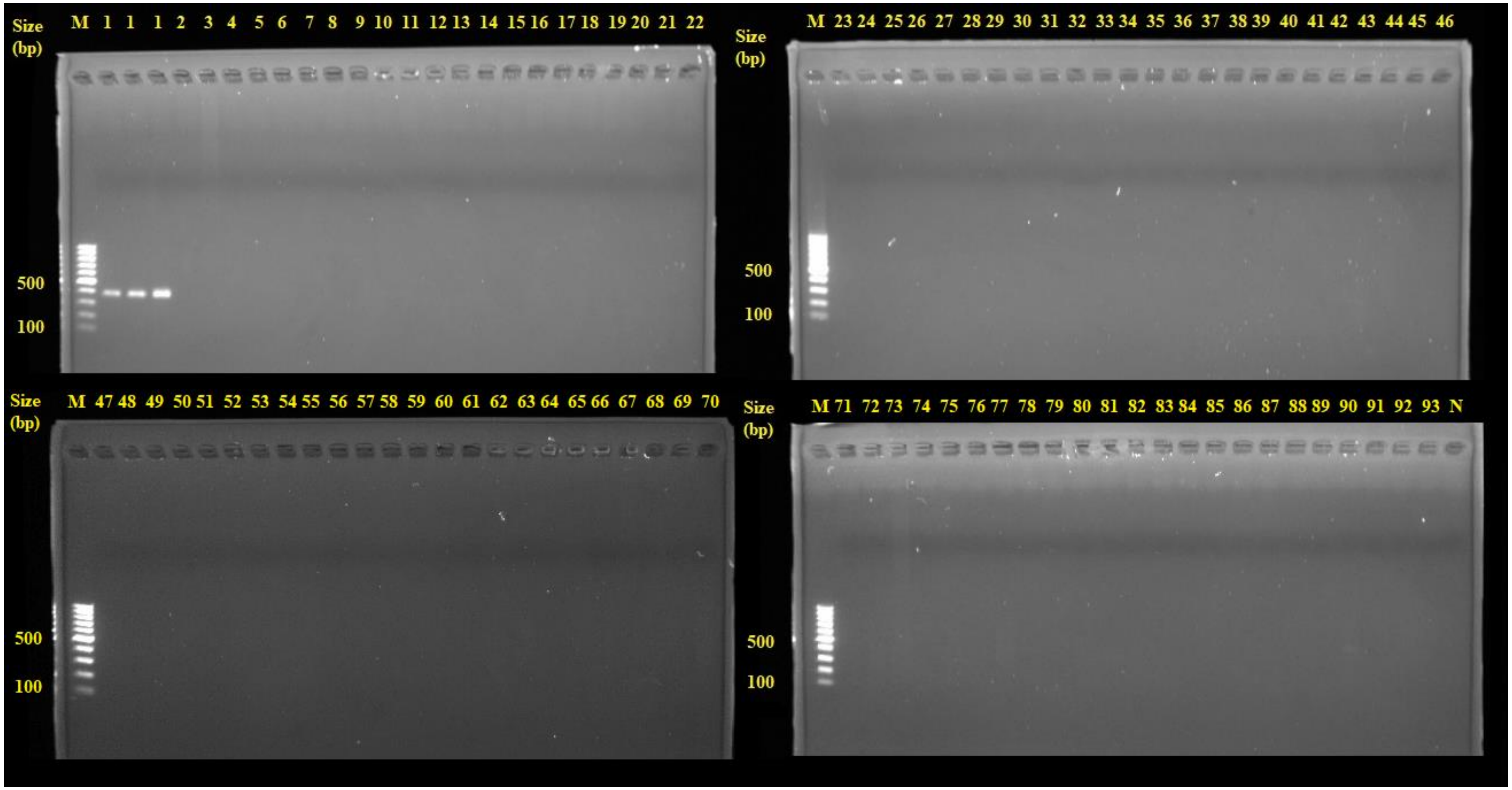

**1 *S. mokarran***	**17 *C. albimarginatus***	**33 *E. brachyurus***	**49 *M. manazo***	**65 *C. amblyrhynchos***	**81 *L. macrorhinus***
**2 *S. lewini***	**18 *C. altimus***	**34 *E. decacuspidatus***	**50 *N. acutiden***	**66 *C. amboinensis***	**82 *M. californicus***
**3 *S. zygaena***	**19 *C. brachyurus***	**35 *E. fusus***	**51 *N. brevirostris***	**67 *C. galapagensis***	**83 *M. canis***
**4 *C. longimanus***	**20 *C. brevipinna***	**36 *E. molleri***	**52 *O. maculatus***	**68 *C. isodon***	**84 *M. henlei***
**5 *L. nasus***	**21 *C. leucas***	**37 *E. pusillus***	**53 *P. xaniurus***	**69 *C. macloti***	**85 *M. lunulatus***
**6 *C. carcharias***	**22 *C. limbatus***	**38 *G. cuvier***	**54 *P. glauca***	**70 *C. porosus***	**86 *M. mosis***
**7 *R. typus***	**23 *C. melanopterus***	**39 *G. sauteri***	**55 *P. habereri***	**71 *Carcharias taurus***	**87 *M. mustelus***
**8 *C. maximus***	**24 *C. obscurus***	**40 *G. cirratum***	**56 *S. pacificus***	**72 *Dalatias licha***	**88 *M. punctulatus***
**9 *C. falciformis***	**25 *C. perezi***	**41 *H. japanica***	**57 *S. tudes***	**73 *D. profundorum***	**89 *R. acutus***
**10 *A. pelagicus***	**26 *C. plumbeus***	**42 *H. perlo***	**58 *S. brevirostris***	**74 *E. blochii***	**90 *R. longurio***
**11 *A. superciliosus***	**27 *C. sorrah***	**43 *I. oxyrinchus***	**59 *S. japonicus***	**75 *G. galeus***	**91 *R. taylori***
**12 *A. vulpinus***	**28 *C. acus***	**44 *I. paucus***	**60 *S. legnota***	**76 *H. australiensis***	**92 *S. laticaudus***
**13 *A. macrorhynchus***	**29 *C. fabricii***	**45 *L. ditropis***	**61 *T. kabeyai***	**77 *H. elongata***	**93 *S. tiburo***
**14 *A. nakayai***	**30 *C. umbratile***	**46 *M. pelagios***	**62 *C. callorynchus***	**78 *H. griseus***	**N Negative Control**
**15 *A. platyrhynchus***	**31 *D. quadrispinosa***	**47 *M. owstoni***	**63 *C. acronotus***	**79 *H. novaezealandiae***	**M 100 bp DNA marker**
**16 *A. marmoratus***	**32 *E. cookei***	**48 *M. griseus***	**64 *C. amblyrhynchoides***	**80 *L. temminckii***	**E Empty**

Figure S14 Amplification results of all 93 species with species-specific PCR primers targeting smooth hammerhead shark *Sphyrna zygaena*.

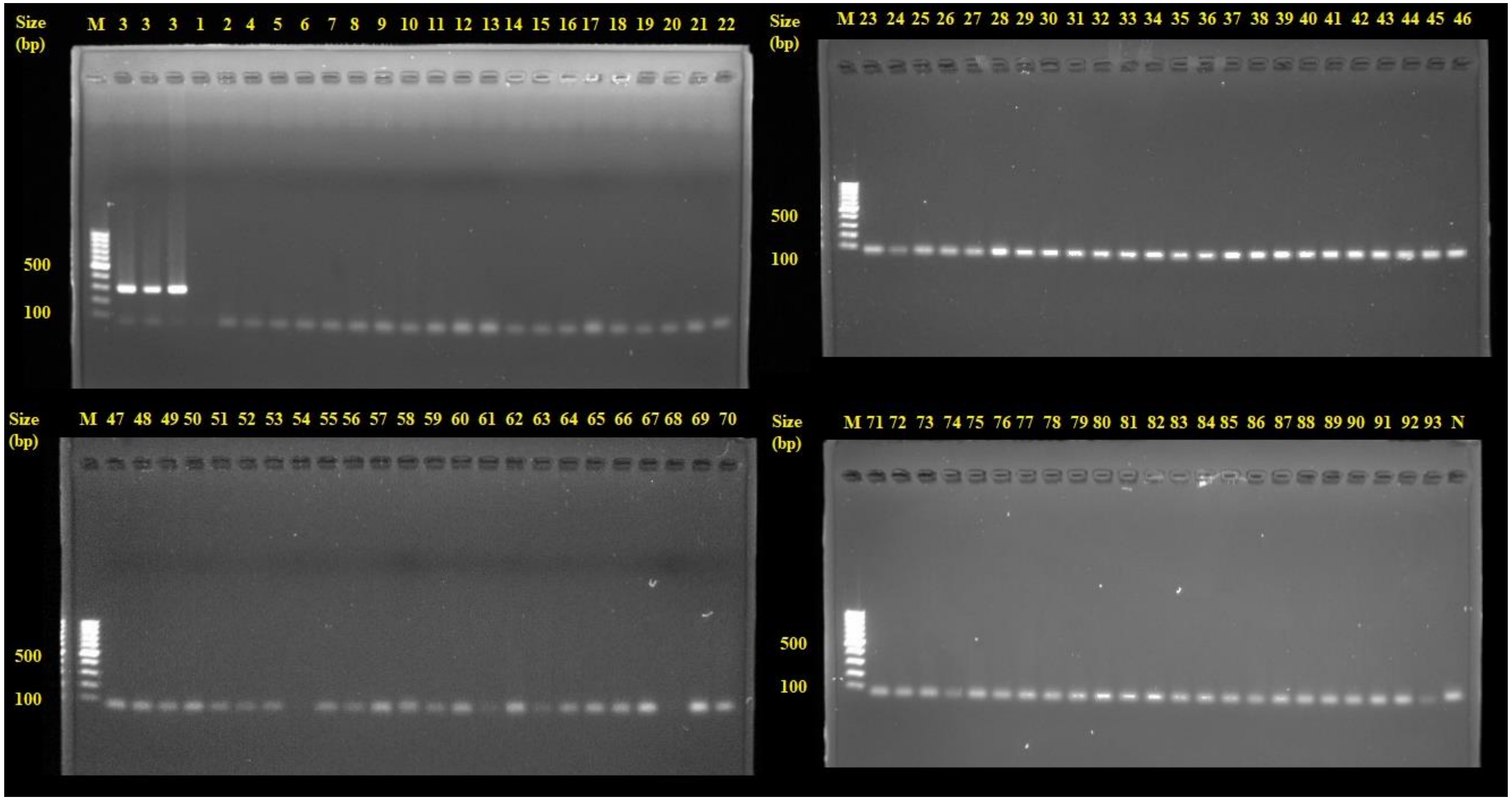

1 ***S. mokarran***
2 ***S. lewini***
3 ***S. zygaena***
4 ***C. longimanus***
5 ***L. nasus***
6 ***C. carcharias***
7 ***R. typus***
8 ***C. maximus***
9 ***C. falciformis***
10 ***A. pelagicus***
11 ***A. superciliosus***
12 ***A. vulpinus***
13 ***A. macrorhynchus***
14 ***A. nakayai***
15 ***A. platyrhynchus***
16 ***A. marmoratus***
17 ***C. albimarginatus***
18 ***C. altimus***
19 ***C. brachyurus***
20 ***C. brevipinna***
21 ***C. leucas***
22 ***C. limbatus***
23 ***C. melanopterus***
24 ***C. obscurus***
25 ***C. perezi***
26 ***C. plumbeus***
27 ***C. sorrah***
28 ***C. acus***
29 ***C. fabricii***
30 ***C. umbratile***
31 ***D. quadrispinosa***
32 ***E. cookei***
33 ***E. brachyurus***
34 ***E. decacuspidatus***
35 ***E. fusus***
36 ***E. molleri***
37 ***E. pusillus***
38 ***G. cuvier***
39 ***G. sauteri***
40 ***G. cirratum***
41 ***H. japanica***
42 ***H. perlo***
43 ***I. oxyrinchus***
44 ***I. paucus***
45 ***L. ditropis***
46 ***M. pelagios***
47 ***M. owstoni***
48 ***M. griseus***
49 ***M. manazo***
50 ***N. acutiden***
51 ***N. brevirostris***
52 ***O. maculatus***
53 ***P. xaniurus***
54 ***P. glauca***
55 ***P. habereri***
56 ***S. pacificus***
57 ***S. tudes***
58 ***S. brevirostris***
59 ***S. japonicus***
60 ***S. legnota***
61 ***T. kabeyai***
62 ***C. callorynchus***
63 ***C. acronotus***
64 ***C. amblyrhynchoides***
65 ***C. amblyrhynchos***
66 ***C. amboinensis***
67 ***C. galapagensis***
68 ***C. isodon***
69 ***C. macloti***
70 ***C. porosus***
71 ***Carcharias taurus***
72 ***Dalatias licha***
73 ***D. profundorum***
74 ***E. blochii***
75 ***G. galeus***
76 ***H. australiensis***
77 ***H. elongata***
78 ***H. griseus***
79 ***H. novaezealandiae***
80 ***L. temminckii***
81 ***L. macrorhinus***
82 ***M. californicus***
83 ***M. canis***
84 ***M. henlei***
85 ***M. lunulatus***
86 ***M. mosis***
87 ***M. mustelus***
88 ***M. punctulatus***
89 ***R. acutus***
90 ***R. longurio***
91 ***R. taylori***
92 ***S. laticaudus***
93 ***S. tiburo***
N **Negative Control**
M **100 bp DNA marker**
E **Empty**

Figure S15 Sensitivity of the CITES-listed shark species PCR assay targeting pelagic thresher shark *Alopias pelagicus* with corresponding target species at the concentration of: lane 1, 10.0 ng/µL; 2, 5.0 ng/µL; 3, 1.0 ng/µL; 4, 0.4 ng/µL; 5, 0.2 ng/µL; 6, 0.1 ng/µL; N, negative control. Sensitivity in terms of limit of detection of each PCR assay for corresponding target species with different concentrations, 10.0 ng/µL, 5.0 ng/µL, 1.0 ng/µL, 0.4 ng/µL, 0.2 ng/µL, and 0.1 ng/µL.

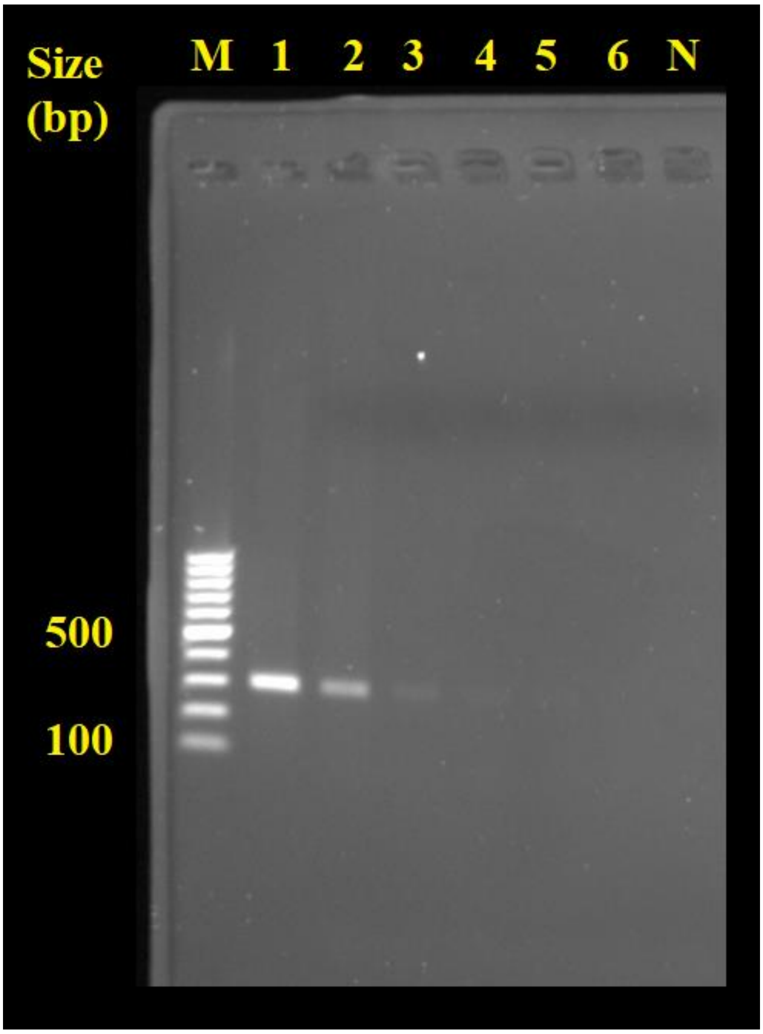

Figure S16 Sensitivity of the CITES-listed shark species PCR assay targeting bigeye thresher shark *Alopias superciliosus* with corresponding target species at the concentration of: lane 1, 10.0 ng/µL; 2, 5.0 ng/µL; 3, 1.0 ng/µL; 4, 0.4 ng/µL; 5, 0.2 ng/µL; 6, 0.1 ng/µL; N, negative control. Sensitivity in terms of limit of detection of each PCR assay for corresponding target species with different concentrations, 10.0 ng/µL, 5.0 ng/µL, 1.0 ng/µL, 0.4 ng/µL, 0.2 ng/µL, and 0.1 ng/µL.

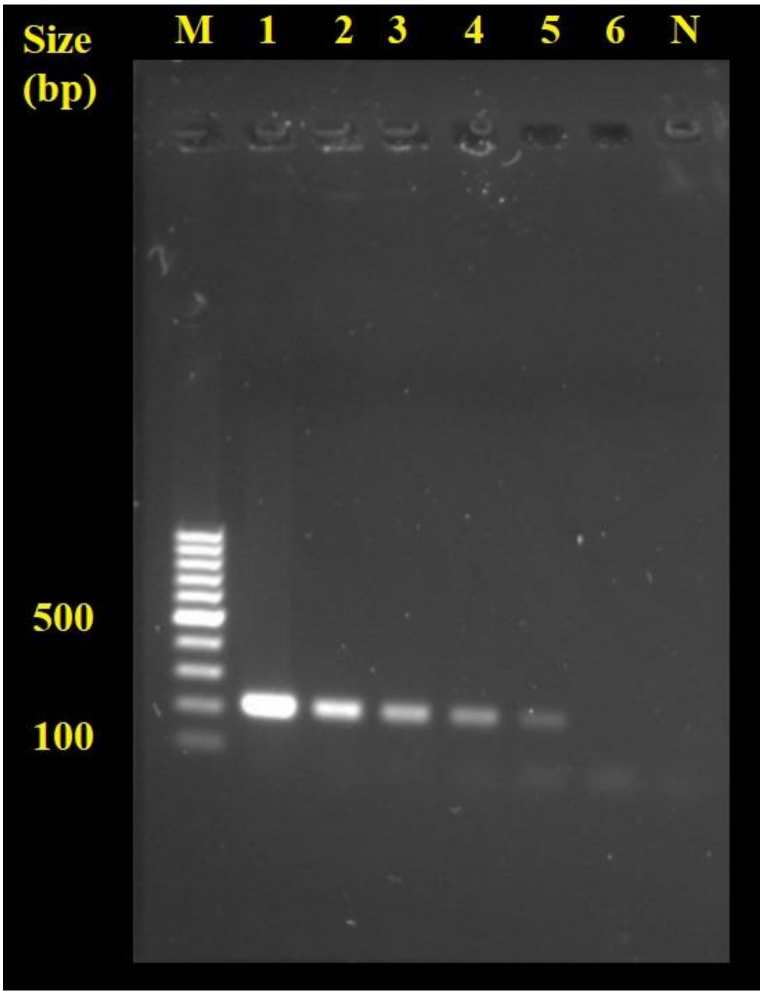

Figure S17 | Sensitivity of the CITES-listed shark species PCR assay targeting common thresher shark *Alopias vulpinus* with corresponding target species at the concentration of: lane 1, 10.0 ng/µL; 2, 5.0 ng/µL; 3, 1.0 ng/µL; 4, 0.4 ng/µL; 5, 0.2 ng/µL; 6, 0.1 ng/µL; N, negative control. Sensitivity in terms of limit of detection of each PCR assay for corresponding target species with different concentrations, 10.0 ng/µL, 5.0 ng/µL, 1.0 ng/µL, 0.4 ng/µL, 0.2 ng/µL, and 0.1 ng/µL.

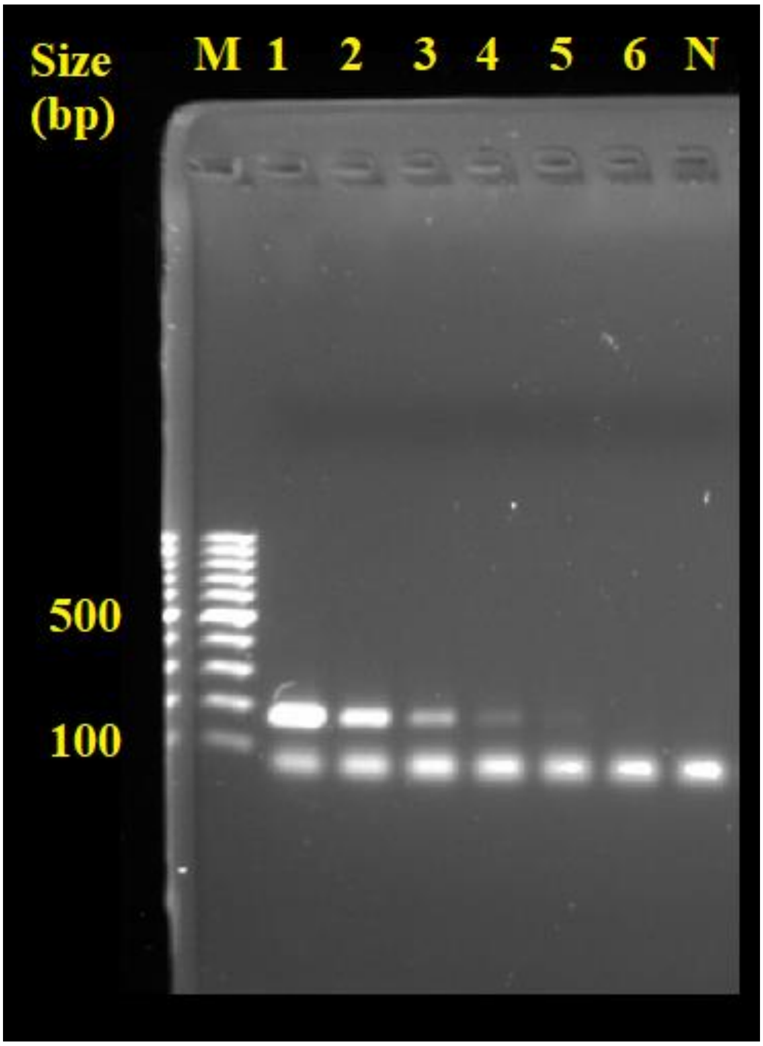

Figure S18 | Sensitivity of the CITES-listed shark species PCR assay targeting great white shark *Carcharodon carcharias* with corresponding target species at the concentration of: lane 1, 10.0 ng/µL; 2, 5.0 ng/µL; 3, 1.0 ng/µL; 4, 0.4 ng/µL; 5, 0.2 ng/µL; 6, 0.1 ng/µL; N, negative control. Sensitivity in terms of limit of detection of each PCR assay for corresponding target species with different concentrations, 10.0 ng/µL, 5.0 ng/µL, 1.0 ng/µL, 0.4 ng/µL, 0.2 ng/µL, and 0.1 ng/µL.

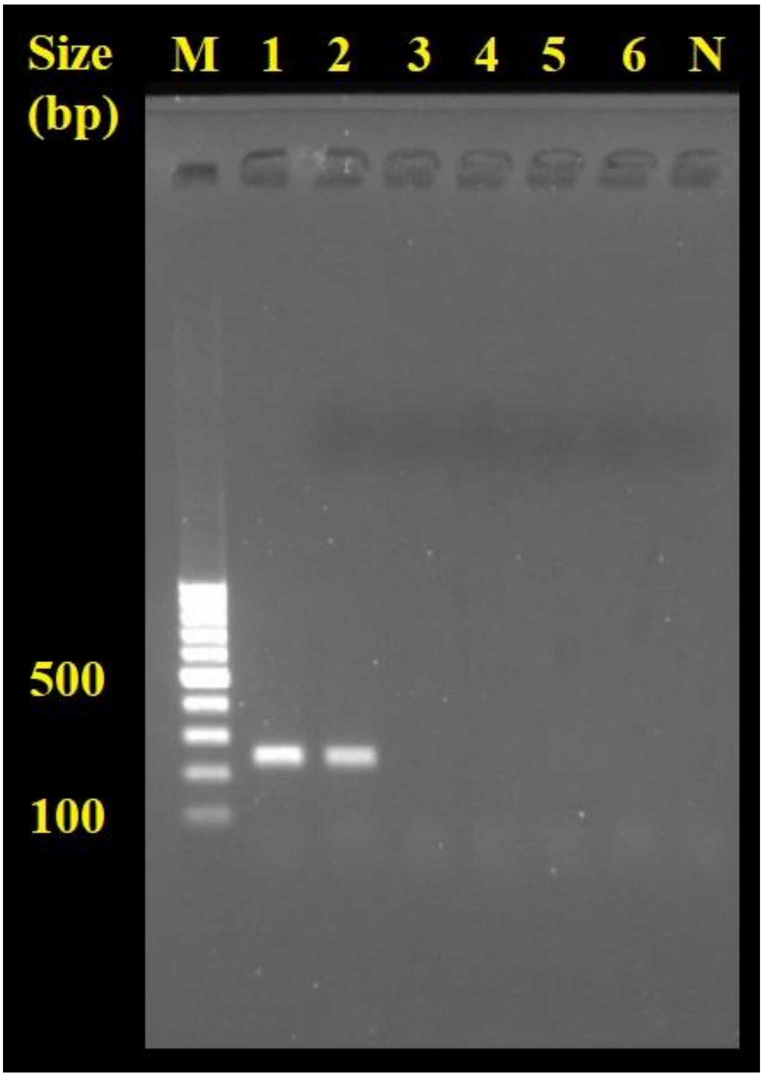

Figure S19 | Sensitivity of the CITES-listed shark species PCR assay targeting silky shark *Carcharhinus falciformis* with corresponding target species at the concentration of: lane 1, 10.0 ng/µL; 2, 5.0 ng/µL; 3, 1.0 ng/µL; 4, 0.4 ng/µL; 5, 0.2 ng/µL; 6, 0.1 ng/µL; N, negative control. Sensitivity in terms of limit of detection of each PCR assay for corresponding target species with different concentrations, 10.0 ng/µL, 5.0 ng/µL, 1.0 ng/µL, 0.4 ng/µL, 0.2 ng/µL, and 0.1 ng/µL.

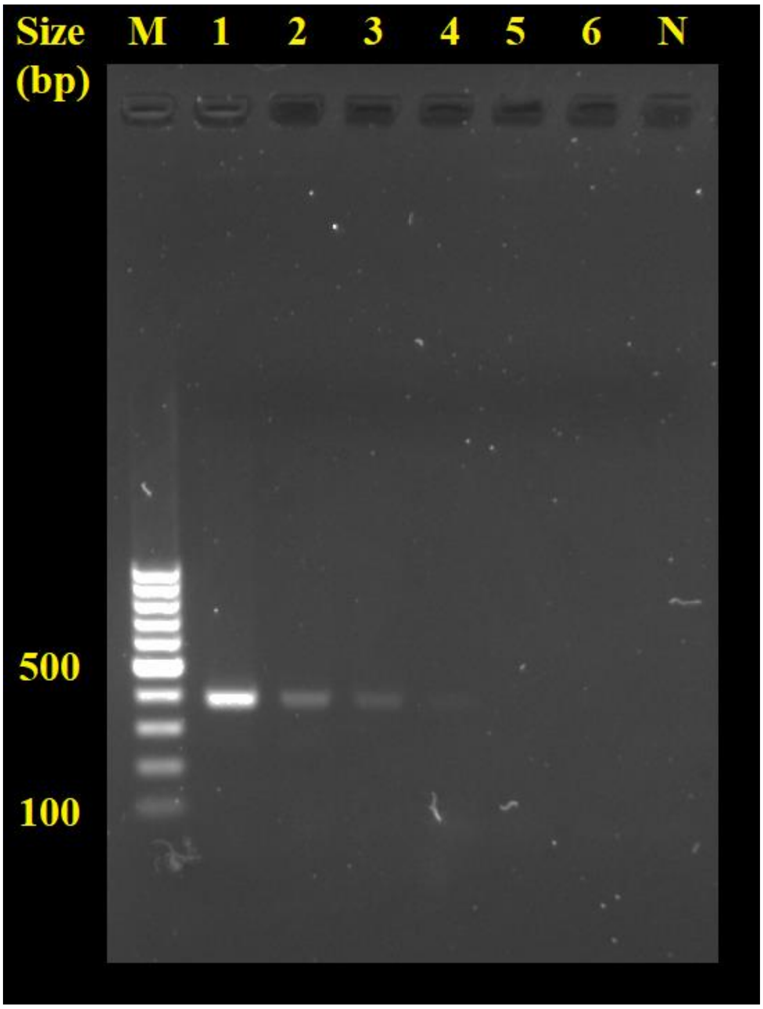

Figure S20 | Sensitivity of the CITES-listed shark species PCR assay targeting oceanic whitetip shark *Carcharhinus longimanus* with corresponding target species at the concentration of: lane 1, 10.0 ng/µL; 2, 5.0 ng/µL; 3, 1.0 ng/µL; 4, 0.4 ng/µL; 5, 0.2 ng/µL; 6, 0.1 ng/µL; N, negative control. Sensitivity in terms of limit of detection of each PCR assay for corresponding target species with different concentrations, 10.0 ng/µL, 5.0 ng/µL, 1.0 ng/µL, 0.4 ng/µL, 0.2 ng/µL, and 0.1 ng/µL.

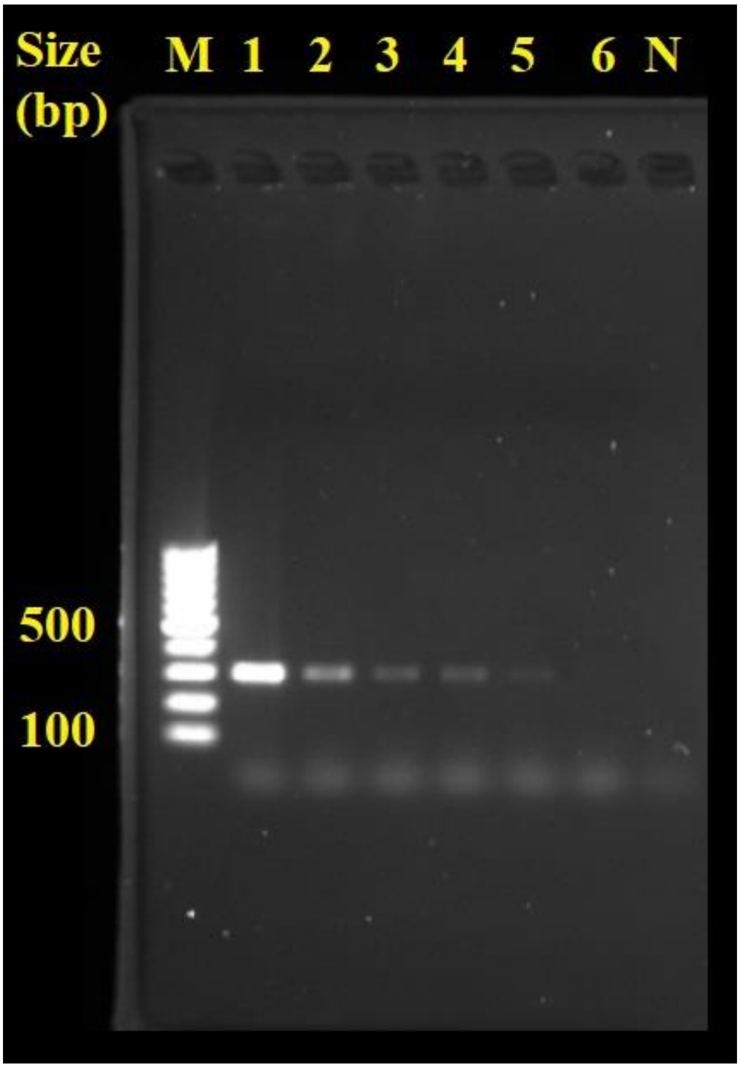

Figure S21 Sensitivity of the CITES-listed shark species PCR assay targeting basking shark *Cetorhinus maximus* with corresponding target species at the concentration of: lane 1, 10.0 ng/µL; 2, 5.0 ng/µL; 3, 1.0 ng/µL; 4, 0.4 ng/µL; 5, 0.2 ng/µL; 6, 0.1 ng/µL; N, negative control. Sensitivity in terms of limit of detection of each PCR assay for corresponding target species with different concentrations, 10.0 ng/µL, 5.0 ng/µL, 1.0 ng/µL, 0.4 ng/µL, 0.2 ng/µL, and 0.1 ng/µL.

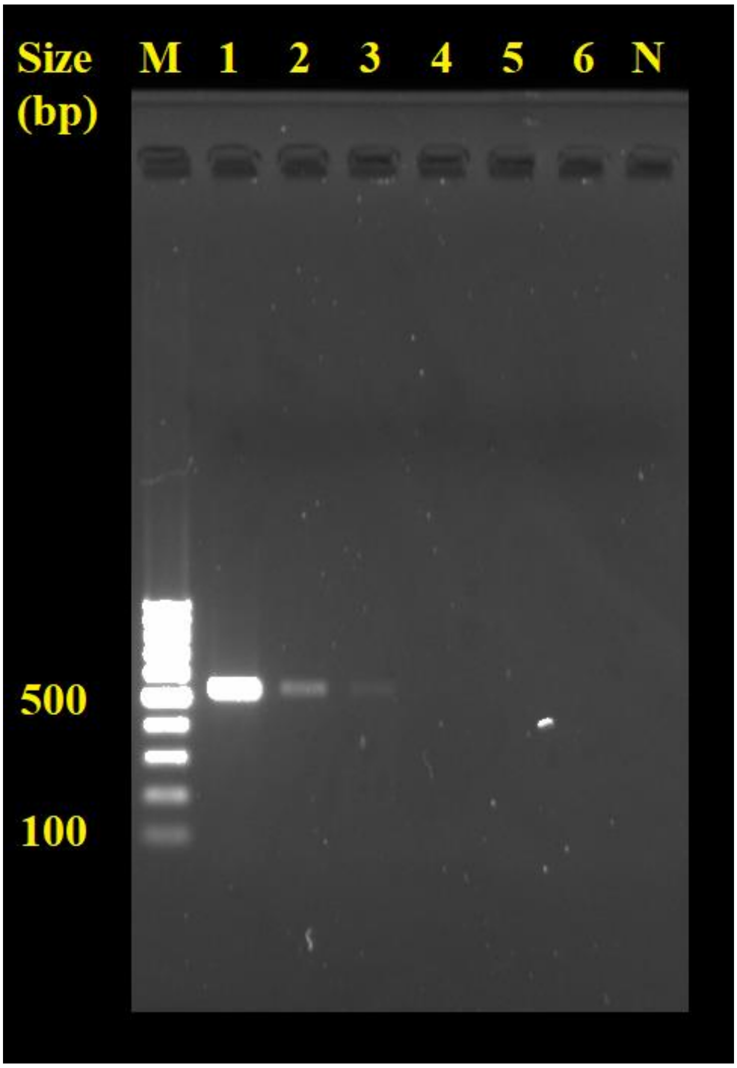

Figure S22 Sensitivity of the CITES-listed shark species PCR assay targeting porbeagle shark *Lamna nasus* with corresponding target species at the concentration of: lane 1, 10.0 ng/µL; 2, 5.0 ng/µL; 3, 1.0 ng/µL; 4, 0.4 ng/µL; 5, 0.2 ng/µL; 6, 0.1 ng/µL; N, negative control. Sensitivity in terms of limit of detection of each PCR assay for corresponding target species with different concentrations, 10.0 ng/µL, 5.0 ng/µL, 1.0 ng/µL, 0.4 ng/µL, 0.2 ng/µL, and 0.1 ng/µL.

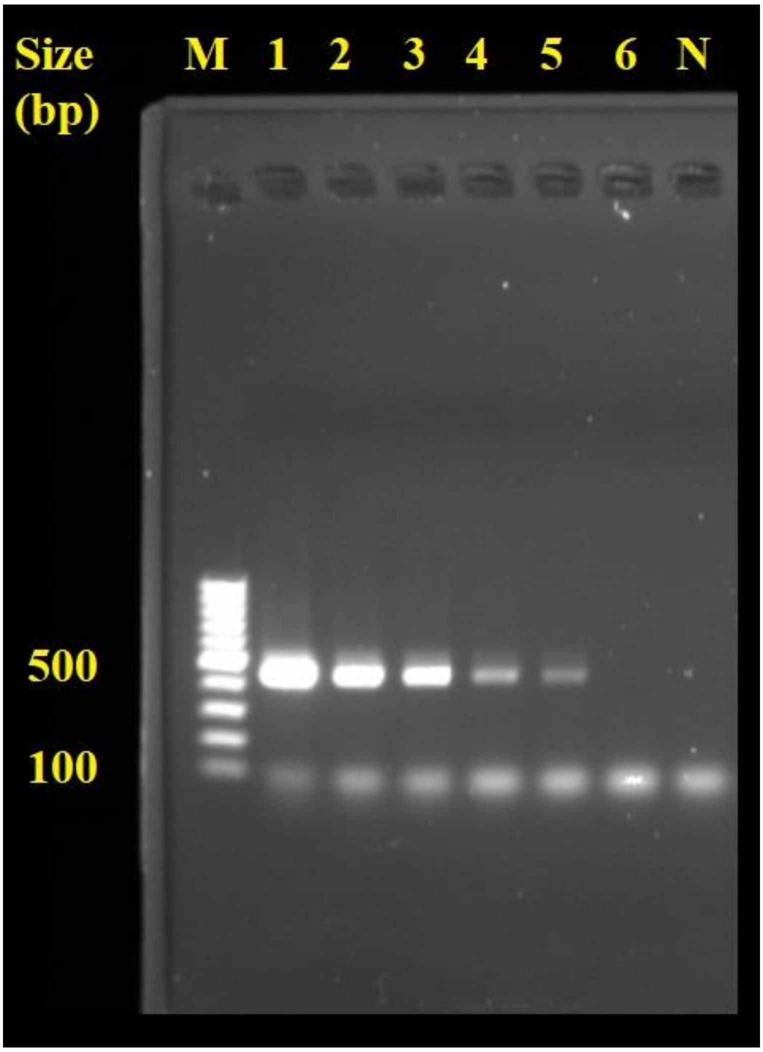

Figure S23 Sensitivity of the CITES-listed shark species PCR assay targeting whale shark *Rhincodon typus* with corresponding target species at the concentration of: lane 1, 10.0 ng/µL; 2, 5.0 ng/µL; 3, 1.0 ng/µL; 4, 0.4 ng/µL; 5, 0.2 ng/µL; 6, 0.1 ng/µL; N, negative control. Sensitivity in terms of limit of detection of each PCR assay for corresponding target species with different concentrations, 10.0 ng/µL, 5.0 ng/µL, 1.0 ng/µL, 0.4 ng/µL, 0.2 ng/µL, and 0.1 ng/µL.

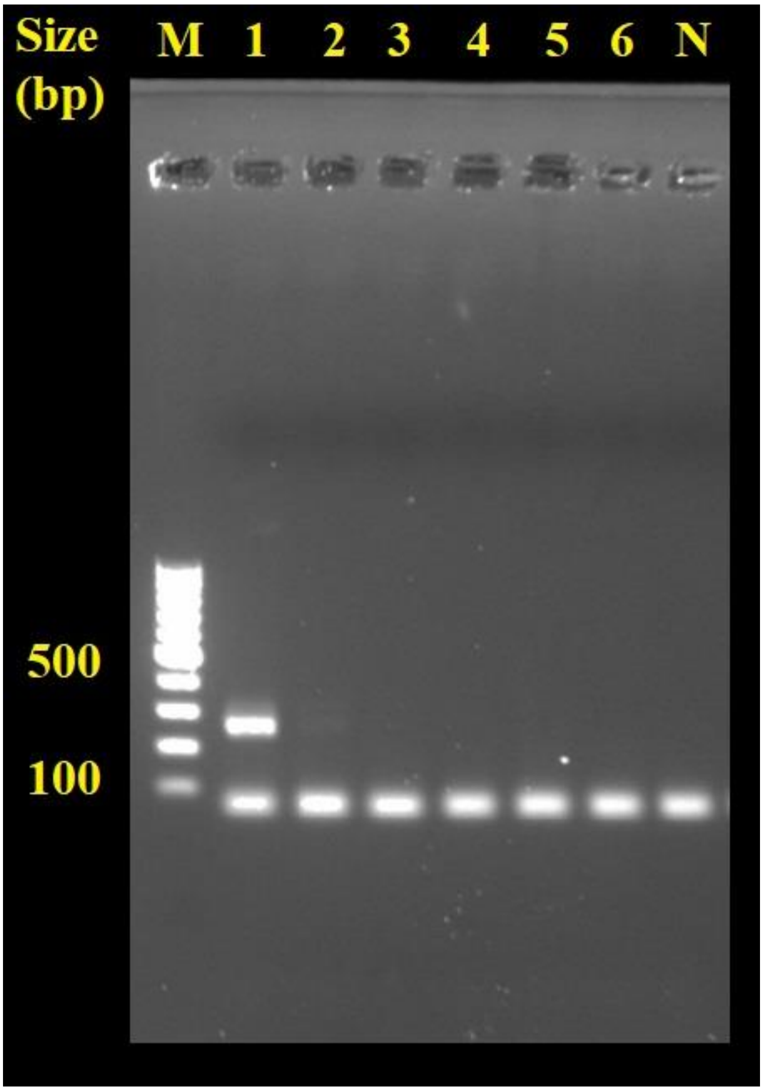

Figure S24 Sensitivity of the CITES-listed shark species PCR assay targeting scalloped hammerhead shark *Sphyrna lewini* with corresponding target species at the concentration of: lane 1, 10.0 ng/µL; 2, 5.0 ng/µL; 3, 1.0 ng/µL; 4, 0.4 ng/µL; 5, 0.2 ng/µL; 6, 0.1 ng/µL; N, negative control. Sensitivity in terms of limit of detection of each PCR assay for corresponding target species with different concentrations, 10.0 ng/µL, 5.0 ng/µL, 1.0 ng/µL, 0.4 ng/µL, 0.2 ng/µL, and 0.1 ng/µL.

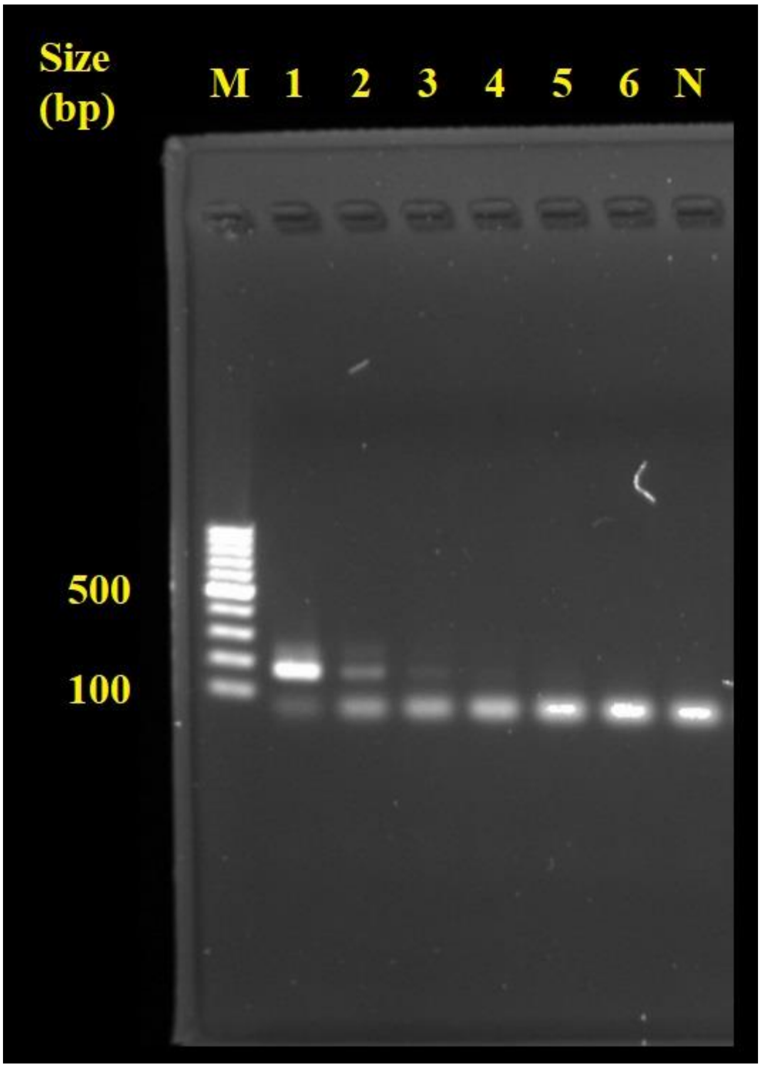

Figure S25 Sensitivity of the CITES-listed shark species PCR assay targeting great hammerhead shark *Sphyrna mokarran* with corresponding target species at the concentration of: lane 1, 10.0 ng/µL; 2, 5.0 ng/µL; 3, 1.0 ng/µL; 4, 0.4 ng/µL; 5, 0.2 ng/µL; 6, 0.1 ng/µL; N, negative control. Sensitivity in terms of limit of detection of each PCR assay for corresponding target species with different concentrations, 10.0 ng/µL, 5.0 ng/µL, 1.0 ng/µL, 0.4 ng/µL, 0.2 ng/µL, and 0.1 ng/µL.

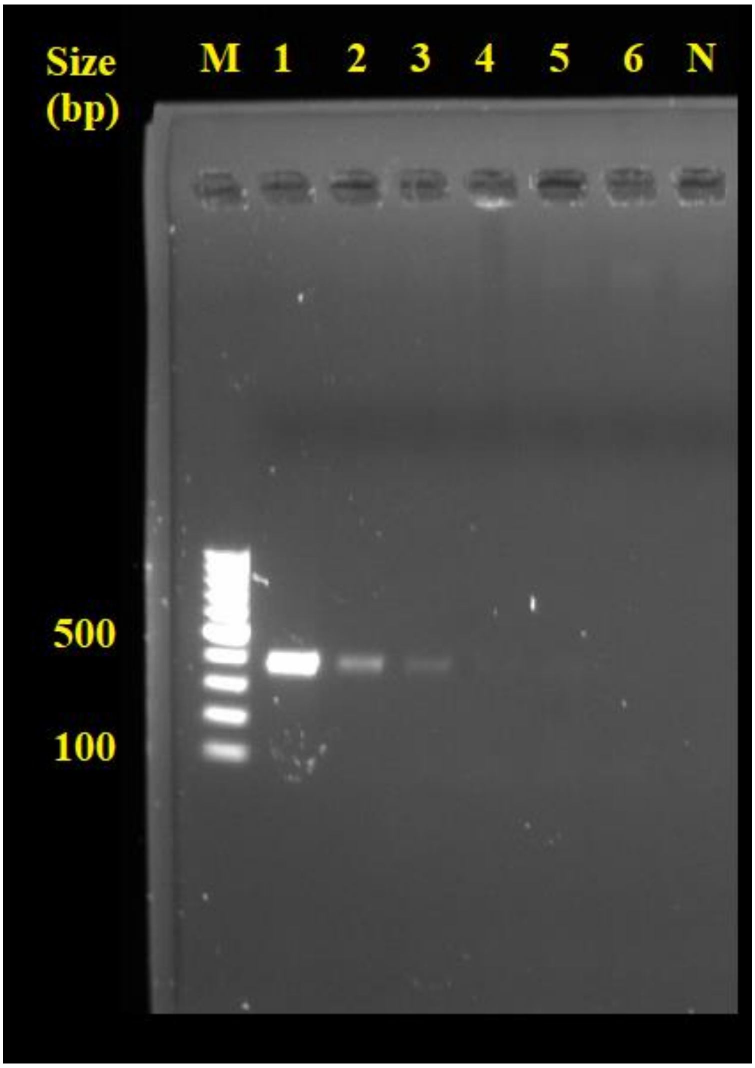

Figure S26 Sensitivity of the CITES-listed shark species PCR assay targeting smooth hammerhead shark *Sphyrna zygaena* with corresponding target species at the concentration of: lane 1, 10.0 ng/µL; 2, 5.0 ng/µL; 3, 1.0 ng/µL; 4, 0.4 ng/µL; 5, 0.2 ng/µL; 6, 0.1 ng/µL; N, negative control. Sensitivity in terms of limit of detection of each PCR assay for corresponding target species with different concentrations, 10.0 ng/µL, 5.0 ng/µL, 1.0 ng/µL, 0.4 ng/µL, 0.2 ng/µL, and 0.1 ng/µL.

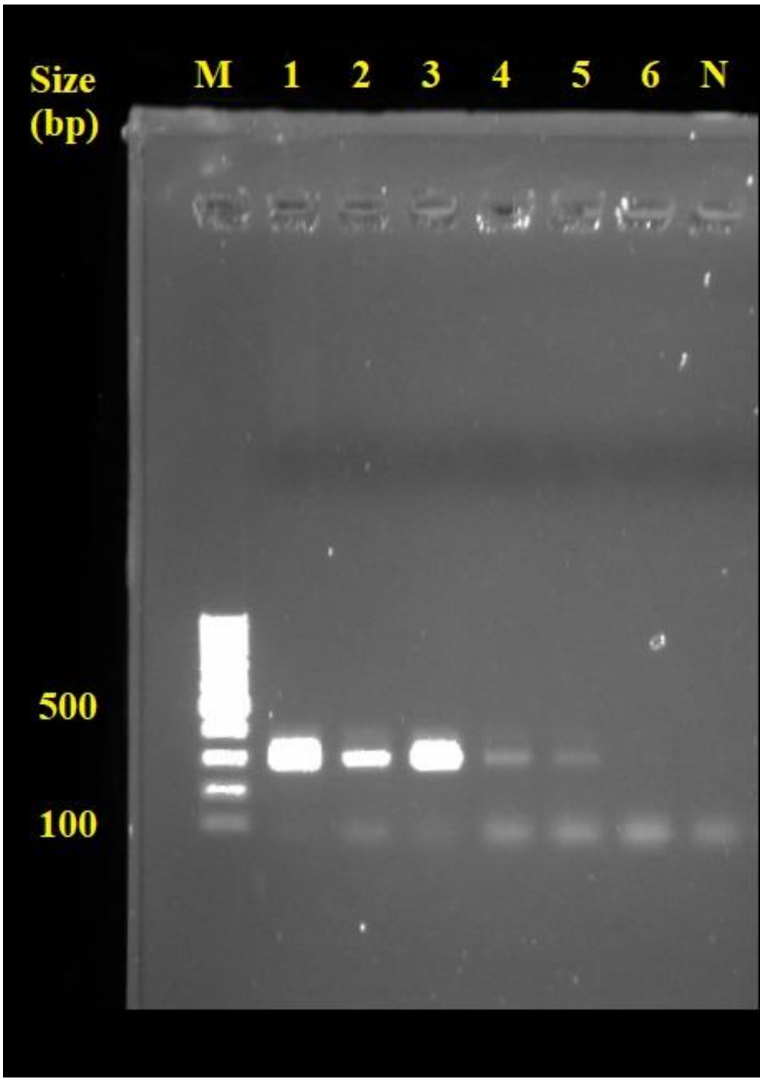

Supplement: Supplementary file 1 — Supplementary Information. [file 41598_2020_61150_MOESM1_ESM.pdf]
